# Exploring immune modulation in osteoarthritis: identifying key biomarkers and the role of PTPRC in regulating immune microenvironment for therapeutic intervention

**DOI:** 10.3389/fimmu.2026.1795075

**Published:** 2026-04-13

**Authors:** Zhengyao Zhang, Yilin Wang, Zhiwen Tan, Xiaohui Yu, Bo Liu

**Affiliations:** 1 Women and Children’s Hospital of Dalian University of Technology, Dalian, China; 2 School of Chemical Engineering, Ocean and Life Sciences, Dalian University of Technology, Panjin, China; 3 Liaoning Key Laboratory of Integrated Circuit and Biomedical Electronic System, Faculty of Medicine, Dalian University of Technology, Dalian, China; 4 Leicester International Institute, Dalian University of Technology, Panjin, China

**Keywords:** bioinformatics, biomarkers, immune microenvironment, natural killer cell, osteoarthritis

## Abstract

**Background:**

Osteoarthritis (OA) is widely recognized as the most prevalent degenerative disorder affecting the joints, representing a major contributor to chronic pain and disability. Despite its high burden, the molecular mechanisms underlying OA pathogenesis remain poorly understood, particularly in the context of immune microenvironment modulation. This study explores the immune-related OA progression mechanisms and investigates potential biomarkers to aid diagnosis and therapeutic intervention.

**Methods:**

Gene expression data from the GEO database were analyzed, differentially expressed genes (DEGs) and OA-associated gene modules were identified using the LIMMA package in combination with weighted gene co-expression network analysis (WGCNA). Gene Ontology (GO) and Kyoto Encyclopedia of Genes and Genomes (KEGG) analyses were conducted on intersecting genes, which were analyzed using the protein-protein interaction network, XGBoost, and Random Forest, identifying core genes. Subsequently, immune cell infiltration was determined through immune cell infiltration analysis and single-cell sequencing analysis. Next, core genes were validated using Mendelian randomization (MR) and Western blotting (WB). Finally, *in vitro* validation confirmed key findings.

**Results:**

A total of 1,171 upregulated DEGs were identified. WGCNA analysis delineated 25 co-expression modules, and the turquoise module emerged as the most strongly related to OA. The PPI network, XGBoost, and Random Forest analysis pinpointed three hub genes: protein tyrosine phosphatase receptor type C (PTPRC), C-X3-C Motif Chemokine Receptor 1 (CX3CR1) and Integrin Subunit Beta 2 (ITGB2). Immune cell infiltration analysis indicates that these key genes exhibit significant associations with immune cells, while single-cell sequencing and GSEA enrichment analysis further suggested their involvement in inflammatory pathways and immune activation. *In vitro* experiments demonstrate that one of the hub genes—PTPRC—alleviates the deterioration of OA through three levels. Virtual knockout in Natural Killer (NK) cells further confirms that PTPRC influences OA by regulating the immune microenvironment.

**Conclusions:**

This study identified three promising biomarkers in OA and certificated that PTPRC plays a pivotal role in alleviating OA progression through immunomodulation, offering a novel intervention pathway for tissue engineering combined with immunomodulatory therapy in OA.

## 1 Introduction

Osteoarthritis (OA) represents the most prevalent chronic rheumatic disorder globally and constitutes a substantial burden on both individual well-being and public health systems (1). OA primarily affects the spine and synovial joints, such as the fingers, knees, and hips (2). The disease is characterized by persistent structural alterations of the joint, such as articular cartilage degradation, sclerosis of the subchondral bone, development of osteophytes, heterogeneous synovial inflammation, degeneration of periarticular ligaments and menisci, and thickening of the joint capsule (3). Currently, recognized therapeutic targets for OA include:MMP13 (4) and ADAM proteins (5). MMP13 plays a central role in OA-related collagen degradation, exhibiting slight upregulation in early-stage cartilage and pronounced expression in the synovium (4). ADAM-8, -9, -12, and -28 are overexpressed in the early stages of OA (5). In addition, pathways such as AMPK signaling (6), Wnt/β-catenin signaling (7), mTOR signaling (8), NF-κB signaling (9) and focal adhesion signaling (10) have been implicated in the development and progression of OA. At present, there are no approved pharmacological therapies capable of modifying OA progression or preventing long-term functional impairment. Apart from non-pharmacological treatments such as acupuncture (11) and physiotherapy (12) which have uncertain efficacy, international guidelines recommend medications that primarily aim to alleviate symptoms and slow tissue damage to delay disease progression (13). The inability to cure OA stems, in part, from the multifaceted complexity of its pathogenesis and the limited understanding of the specific mechanisms underlying relevant signaling pathways (14). Therefore, gaining a deep understanding of the signaling pathways and therapeutic targets involved in the development of OA is crucial.

The advent of high-throughput techniques (15) and gene microarray technology (16) has made bioinformatics an indispensable tool for effectively identifying disease targets (17, 18). More and more researchers have introduced machine learning (ML) (19) to address a wide range of challenges in the biomedical field. Meanwhile, Mendelian randomization (MR) leverages single nucleotide polymorphisms (SNPs) as instrumental variables to infer causal associations between exposures and outcomes (20), enabling rapid and reliable inference of potential causal links between diseases and symptoms. Moreover, the advancement of single-cell technology (21) has revolutionized how researchers study biological systems. Combining bioinformatics approaches with single-cell sequencing, machine learning, and MR provides a robust framework for enhancing the precision, reliability, and predictive power of disease diagnosis.

**Figure 1:**
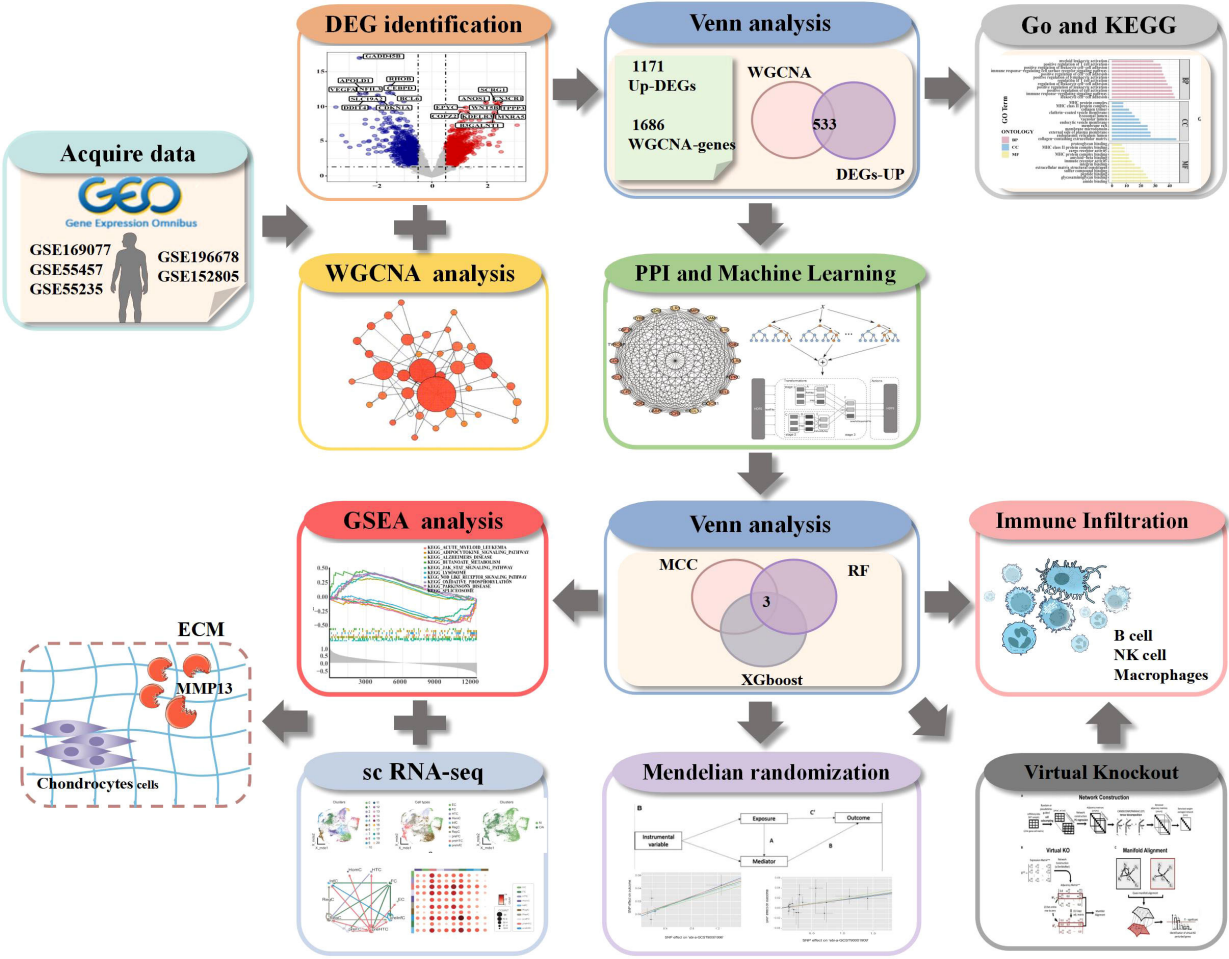
Targets Identification flowchart.

This study sought to uncover key genes involved in OA pathogenesis and to identify potential therapeutic targets using an integrated bioinformatics approach. Gene expression datasets from patients with OA were retrieved from three independent Gene Expression Omnibus (GEO) cohorts. Differential expression analysis, together with weighted gene co-expression network analysis (WGCNA), was subsequently performed to screen for candidate hub genes. followed by enrichment analysis to uncover pathways potentially related to OA. To identify more robust biomarkers for OA treatment, hub genes associated with OA (PTPRC, ITGB2 and CX3CR1) were screened using a combination of two ML algorithms, XGBoost and Random Forest (RF), in conjunction with the protein-protein interaction (PPI) network. Together, these genes highlight the immune and inflammatory pathways in OA, offering novel therapeutic targets beyond traditional cartilage degradation mechanisms. Additionally, through single-cell sequencing and immune infiltration analysis, researchers investigated interactions among chondrocyte subpopulations in OA and the relationship between pivotal genes and immune cells. The results revealed associations between pivotal genes and various immune cells, such as natural killer (NK) cells. Finally, through a series of *in vitro* experiments, the crucial role of the target protein PTPRC in mitigating the release of inflammatory factors and inhibiting extracellular matrix degradation in OA was confirmed. Virtual knockout technology revealed potential alterations in the immune microenvironment following PTPRC knockdown. This provides insights for exploring cytokine-regulated immune environments (**Figure 1**).

## 2 Materials and methods

### 2.1 Acquisition of microarray expression data

Publicly available OA microarray datasets were collected from the NCBI GEO database (https://www.ncbi.nlm.nih.gov/geo/) (22), using “osteoarthritis” as the search keyword.GSE169077, GSE55457, GSE46750 (23) and GSE55235 (24) —were deemed eligible. The clinical characteristics of the samples included in the dataset are presented in the Supplementary Materials (**Supplementary Table 1**). Additionally, the single-cell dataset GSE196678 (25) included four OA samples with 26,379 cells, while the GSE152805 dataset (26) contained three OA samples (11,579 chondrocytes from the diseased medial side) and three normal samples (14,613 chondrocytes from the relatively unaffected lateral side of the tibial cartilage).

### 2.2 Differential expression and WGCNA were conducted to identify genes of interest

To identify genes showing significant differences between OA and healthy samples within a large dataset, differential gene analysis and WGCNA network analysis were employed. Differential gene expression was analyzed using the “limma” package in R. The “GEOquery” package was used to preprocess the expression matrix, followed by network construction using “WGCNA” package (27) to authenticate co-expressed gene modules.

### 2.3 Enrichment analysis

To investigate the biological functions and signaling pathways associated with candidate hub genes, the overlapping genes between differentially expressed genes (DEGs) and co-expression modules were subjected to functional enrichment analysis. Gene Ontology (GO) and Kyoto Encyclopedia of Genes and Genomes (KEGG) pathway analyses were conducted using the clusterProfiler package (28). Results were considered significant (P-value < 0.05).

### 2.4 Detection of hub genes

#### 2.4.1 Construction of PPI network

First, a PPI network was constructed using the STRING database (https://cn.string-db.org/), with interactions between selected genes predicted using a confidence score threshold of 0.4. The rationale is to identify highly connected genes that may serve as central regulators in OA. This network was then visualized using Cytosccape. The CytoHubba plugin was employed to rank nodes within the network based on the Maximum Cluster Centrality (MCC) method, thereby identifying the top 20 genes.

#### 2.4.2 Selection of genes using ML algorithms

Two ML algorithms, RF (29) and XGBoost (30), were applied to rank feature importance and select key genes. These methods were chosen because they can handle high-dimensional data and assess the predictive importance of each gene. Detailed training procedures, cross-validation, and parameter settings are provided in **Supplementary Material**.

#### 2.4.3 Identification of hub genes

The top 20 genes identified from the RF model, XGBoost model, and PPI network were intersected to identify hub genes associated with OA. An online tool (Draw Venn Diagram (ugent.be)) was used to generate a Venn diagram for analyzing overlapping DEGs.

### 2.5 Validation of hub genes

#### 2.5.1 Independent dataset validation

The GSE98918 dataset (31) was downloaded from the GEO database for validation. Differences in core gene expression between OA samples and normal samples were analyzed. P-values < 0.05 were considered statistically significant. Results were visualized using the “ggplot2” package.

#### 2.5.2 MR analysis

MR analysis was performed using the “TwoSampleMR” package (32) in R. MR analysis was performed to explore potential causal relationships between hub genes and OA. This method was selected to provide evidence for causal associations beyond correlation. Detailed thresholds, SNP selection criteria, and software parameters are provided in **Supplementary Materials**.

#### 2.5.3 Western blot analysis

Western blot was performed to validate the protein expression of hub genes in OA and normal samples. The rationale is to confirm that transcript-level changes are reflected at the protein level. Experimental details such as antibody concentrations are included in **Supplementary Materials**.

### 2.6 Immune cell infiltration linked to hub genes expression

The “GSVA” package in R was employed to quantify immune cell-associated transcriptional features and investigate their relationship with key gene expression. This analysis aids in exploring potential underlying interactions between key genes and factors within the immune microenvironment (33).

### 2.7 Gene set enrichment analysis

To predict the potential functions of target genes, single-gene GSEA was conducted using the “clusterProfiler” package (34). In the MsigDB database, the criteria for significantly enriched gene sets are: P < 0.05, FDR q-value < 0.05.

### 2.8 Single-cell sequencing analysis

The GSE196678 dataset (25) was used to study the association between OA and the immune-related microenvironmental factors. This approach allows validation of hub gene expression at the single-cell level and provides insight into immune cell involvement. For further analysis of chondrocytes, the GSE152805 dataset was processed to remove batch effects using Harmony and subjected to similar preprocessing steps. The scCODA model was employed to explore cell-type distributions between OA and normal groups. The “cellchat” package (35) was used to estimate intercellular interaction levels among subpopulations based on the CellPhoneDB database.

### 2.9 Solation and culture of primary human chondrocytes

Digest the partial cartilage from the knee joint of patients who have undergone knee replacement surgery to extract human chondrocytes. The yellowish, uneven areas on the surface are considered inflammatory cells, while the surrounding smooth, white cartilage is regarded as normal cells. The clinical characteristics of the samples involved are presented in the supplementary materials (**Supplementary Table 1**).

### 2.10 Cell viability assay

To investigate the effect of PTPRC on chondrocyte proliferation, a proliferation assay was introduced. Chondrocyte proliferation was assessed using the Cell Counting Kit-8 (CCK-8) assay.

### 2.11 Gene knockdown

To investigate the biological role of PTPRC in OA, we employed gene knockdown to conduct functional perturbation experiments. Seed chondrocytes into 6-well plates 16 hours in advance to ensure cells are in the logarithmic growth phase with approximately 50% confluence at the time of transfection. The working concentration of si-PTPRC was set at 80 nM (2 mL cell culture medium + 8 μL siRNA + 42.5 μL buffer + 7.5 μL Plus per well). The working solution was added to the 6-well plate, which was then gently swirled to ensure uniform distribution. The plate was subsequently incubated in a 37 °C constant-temperature incubator for 48 hours.

### 2.12 Enzyme-linked immunosorbent assay and cell immunofluorescence staining

To investigate changes in chondrocyte phenotype following PTPRC knockdown, ELISA analysis and immunofluorescence staining experiments were employed. The culture supernatants from cells subjected to different treatments were collected, the levels of interleukin-1β (IL-1β) and interleukin-6 (IL-6) in the supernatants were quantified using ELISA kits (JL14113-96T, JL10208-96T, Jonlnbio, China), with procedures performed according to the manufacturer’s instructions.

### 2.13 Virtual knock-out in PTPRC

To simulate the impact of PTPRC on gene regulatory networks, virtual knockout technology was employed. This analysis aids in predicting the potential regulatory roles of hub genes within the OA immune microenvironment. The virtual knockdown of PTPRC was carried out through the scTenifoldKnk tool (36), utilizing single-cell RNA sequencing data. We selected the single-cell transcriptome dataset GSE256137, normalized and preprocessed it. By simulating the knockdown state through approximate node deletion of PTPRC, unsupervised virtual knockdown was achieved, enabling the identification of genes exhibiting significantly altered regulatory relationships due to PTPRC knockdown.

## 3 Results

### 3.1 Screening of 2,022 DEGs in OA

**Figure 2:**
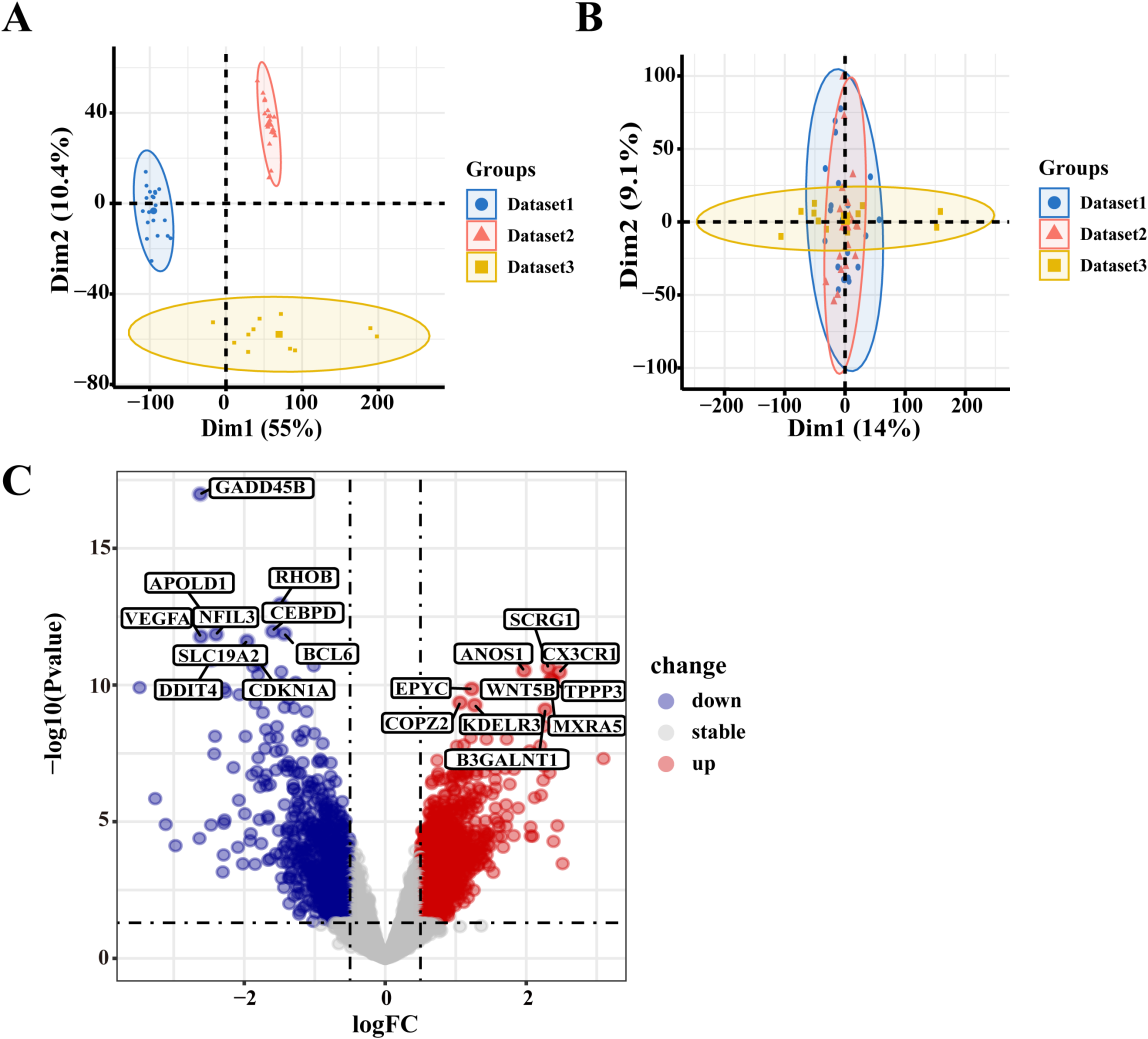
Identification of DEGs. **(A)** Batch effects observed in datasets before correction; **(B)** Integration of the datasets after batch effect correction; **(C)** Volcano diagram illustrating the DEGs between the OA and normal groups.

The datasets GSE55457, GSE169077, and GSE55235 were obtained from the GEO database, encompassing data from 26 OA patients and 25 normal controls. After batch effect removal, the batch effects were significantly mitigated, and the samples from the three datasets were successfully integrated for subsequent analyses (**Figures 2A, B**). Compared to normal controls, the transcriptomic profiles of OA patients exhibited distinct differences. 2,022 DEGs were identified, comprising 851 downregulated and 1,171 upregulated genes (**Supplementary Table 2**). The top 10 genes showing the most significant upregulation and downregulation were further illustrated in **Figure 2C**.

### 3.2 Construction of weighted correlation network for OA and identification of 1,686 co-expressed genes

**Figure 3:**
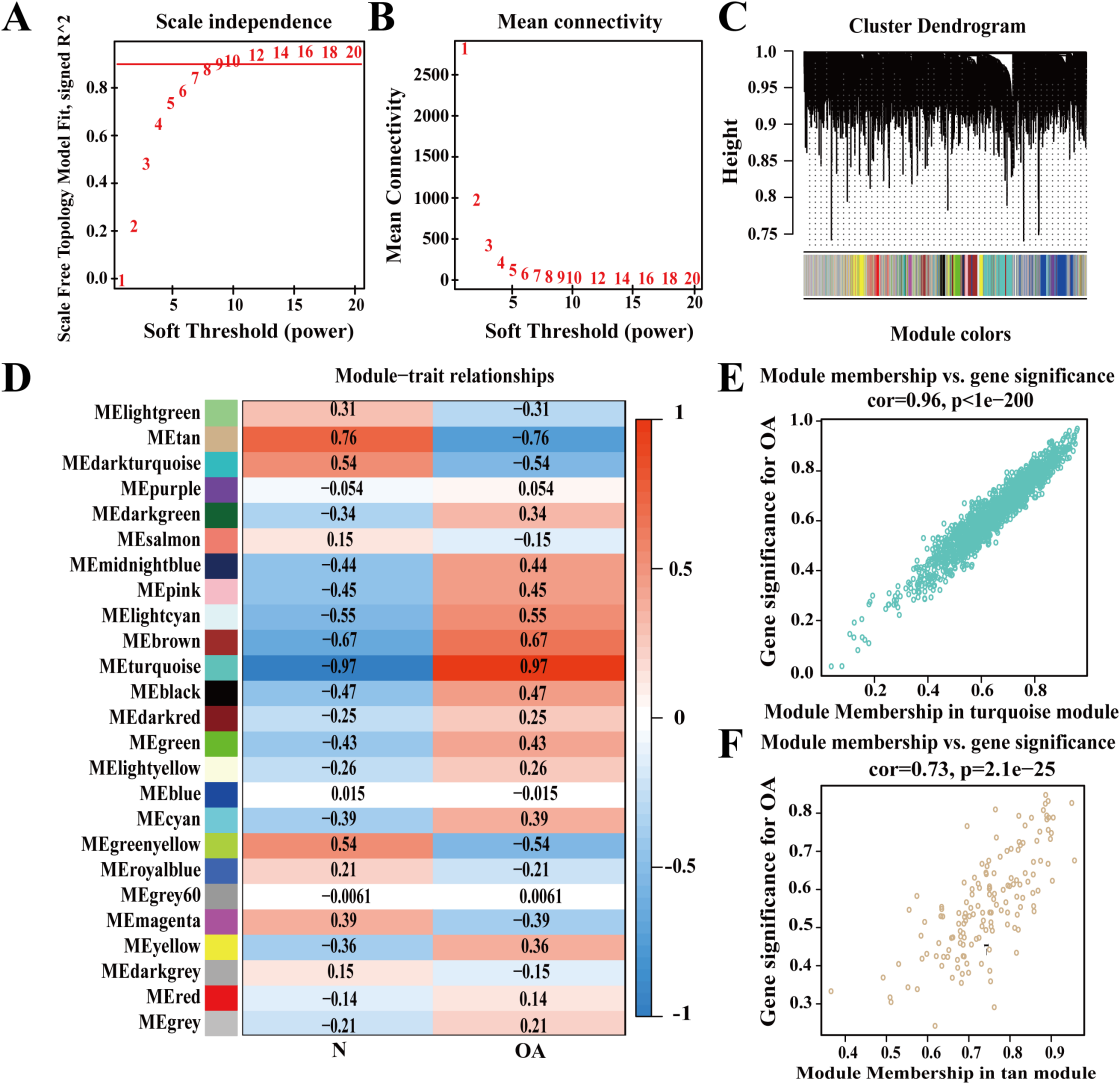
Identification of Co-Expressed Genes. **(A)** Scale-free topology fit index for various soft threshold powers; **(B)** Average connectivity for various soft threshold powers; **(C, D)** Scale-free network divided into 25 gene modules based on correlation; **(E)** Scatterplot of MM versus GS in the turquoise module; **(F)** Scatterplot of MM versus GS in the tan module.

To further identify key genes associated with OA, a WGCNA analysis was performed on OA and normal samples. A scale-free network was constructed using the preprocessed expression matrix. The soft threshold was set to 12 to ensure scale-free topology of the network, with R² = 0.905, indicating high average connectivity (**Figures 3A, B**). Module signature genes represent the overall gene expression levels within each module. These gene signature values undergo computational processing and are subsequently clustered based on their correlation. Twenty-five distinct gene modules exhibiting similar co-expression patterns were identified for further analysis (**Figures 3C, D**). Correlations between module eigengene (ME) values and clinical traits were analyzed to identify clinically significant modules. A positive correlation was viewed between the turquoise module and OA, whereas a negative correlation was noted with normal samples, while the tan module showed the opposite trend, being negatively correlated with OA and positively correlated with normal samples. Scatterplots of module membership (MM) versus gene significance (GS) revealed a positive correlation between MM and GS (**Figures 3E, F**), indicating that genes highly associated with clinical traits also play critical roles within the key modules. A total of 1,686 genes from these modules were identified (**Supplementary Table 3**).

### 3.3 Integration of intersecting genes and enrichment analysis

**Figure 4:**
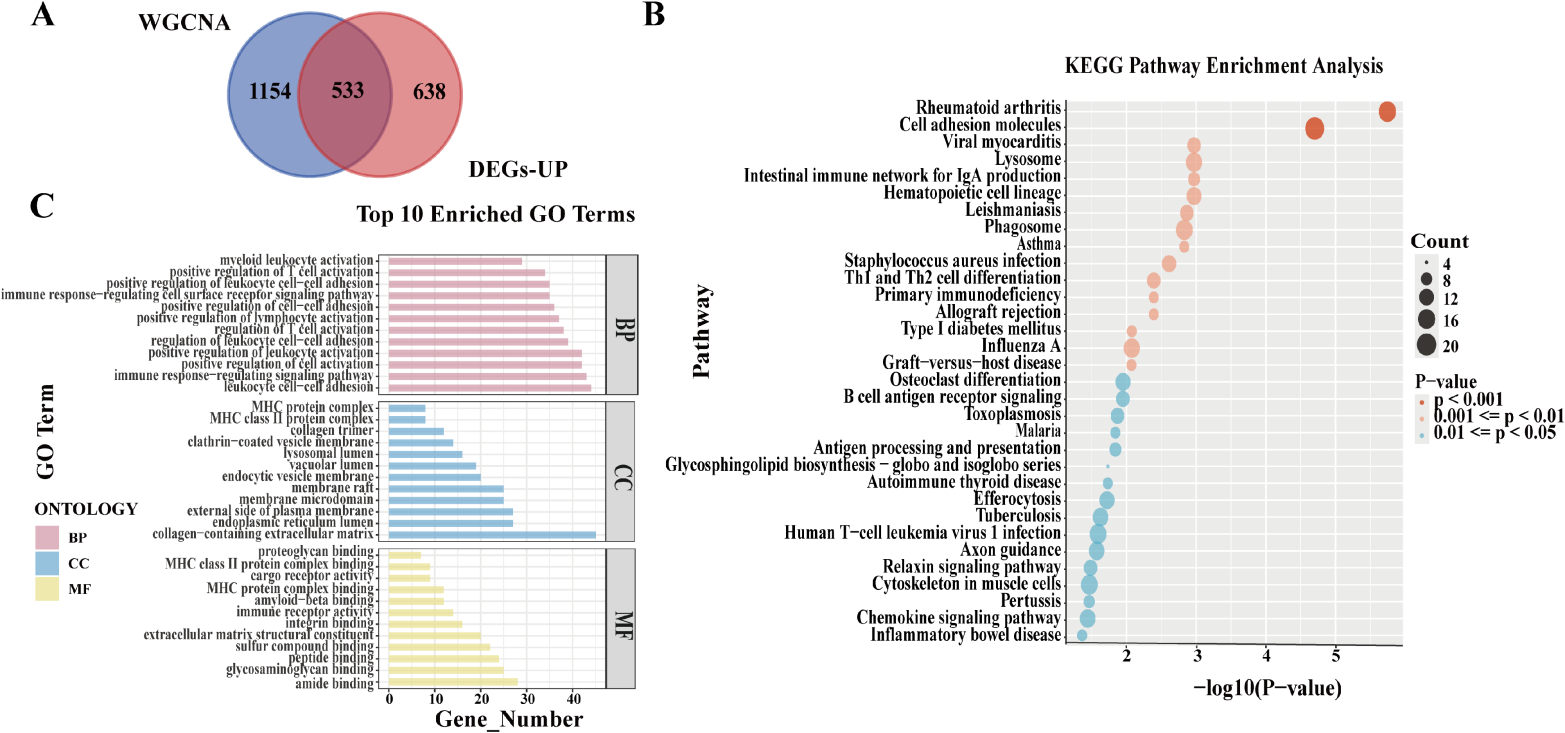
Intersection Genes and Their Enrichment Analysis. **(A)** Intersection genes derived from DEGs and co-expressed genes; **(B)** KEGG pathway analysis of the intersection genes; **(C)** GO pathway analysis of the intersection genes.

The upregulated differentially expressed genes and co-expressed genes were compared, identifying 533 genes in common (**Figure 4A**). GO and KEGG analysis were subsequently performed on these 533 genes. In the KEGG analysis, several enriched pathways were identified in OA compared to normal controls. These pathways included phagosome, lysosome, rheumatoid arthritis, B cell receptor (BCR) signaling pathway, arachidonic acid metabolism, and osteoclast differentiation (**Figure 4B**). In the GO analysis, biological process (BP) enrichment was primarily associated with cell adhesion, cell activation, regulation of immune response signaling pathways, and leukocyte proliferation. Molecular function (MF) enrichment was related to amide binding, immune receptor activity, and oxidoreductase activity. Cellular component (CC) enrichment was linked to lysosomal endoplasmic reticulum and collagen-containing extracellular matrix (**Figure 4C**). The above results suggest that the intersection genes may promote chondrocyte apoptosis through immune regulatory signaling pathways, thereby accelerating the progression of OA.

We also calculated the intersection between downregulated differentially expressed genes and co-expressed genes and performed pathway enrichment analysis. Our analysis of downregulated DEGs revealed significant enrichment in several immune-related pathways, such as MAPK signaling, IL-17 signaling, and FoxO signaling, which are involved in immune regulation and cellular homeostasis. While these pathways are typically associated with immune response control, their downregulation suggests that immune regulatory mechanisms are impaired in OA, contributing to immune network dysregulation. These findings further support the concept of dual remodeling of the immune network in OA, where both immune activation and immune regulation are disrupted (**Supplementary Figure 1**). Since upregulated genes are more likely to reflect immune activation, we selected the 533 differentially co-expressed genes that were up-regulated for subsequent experiments.

### 3.4 Identification of hub genes using the combination of PPI network and ML algorithms

**Figure 5:**
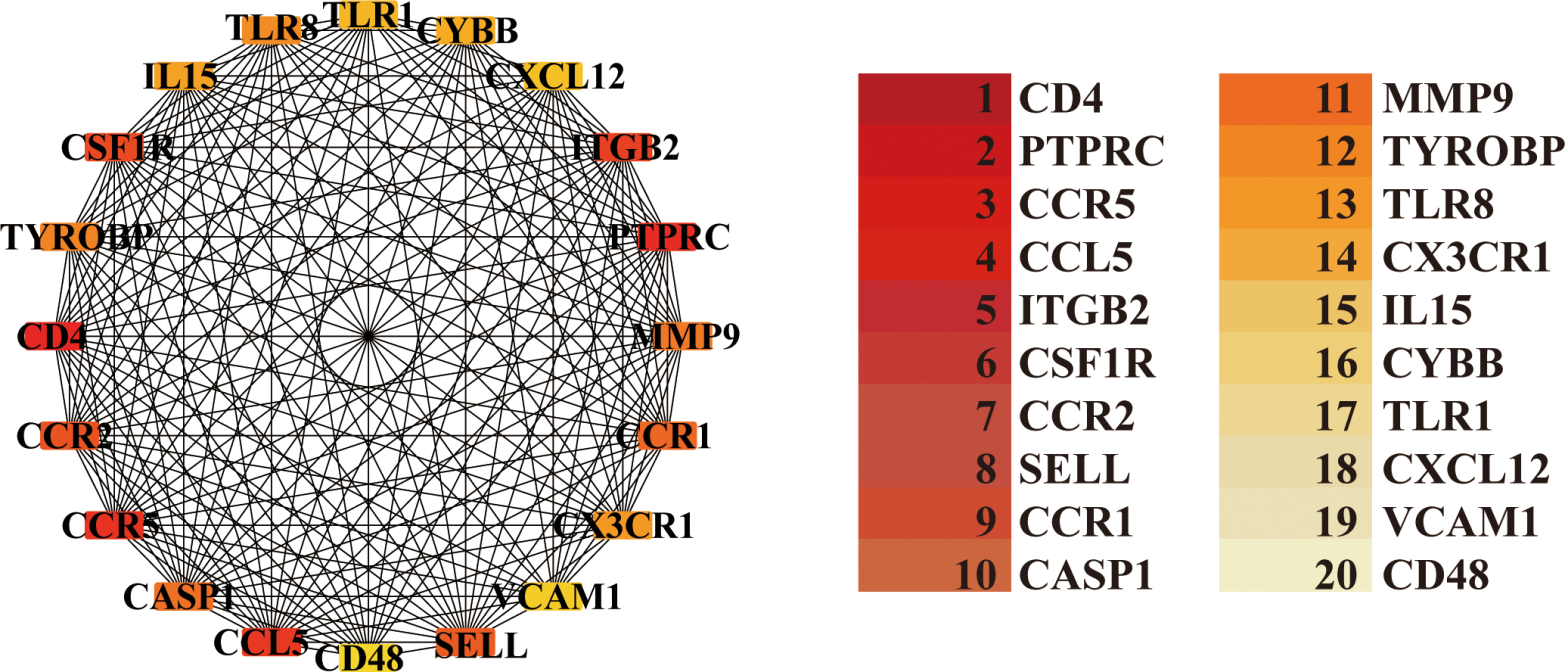
The top 20 nodes calculated by the PPI network.

**Figure 6:**
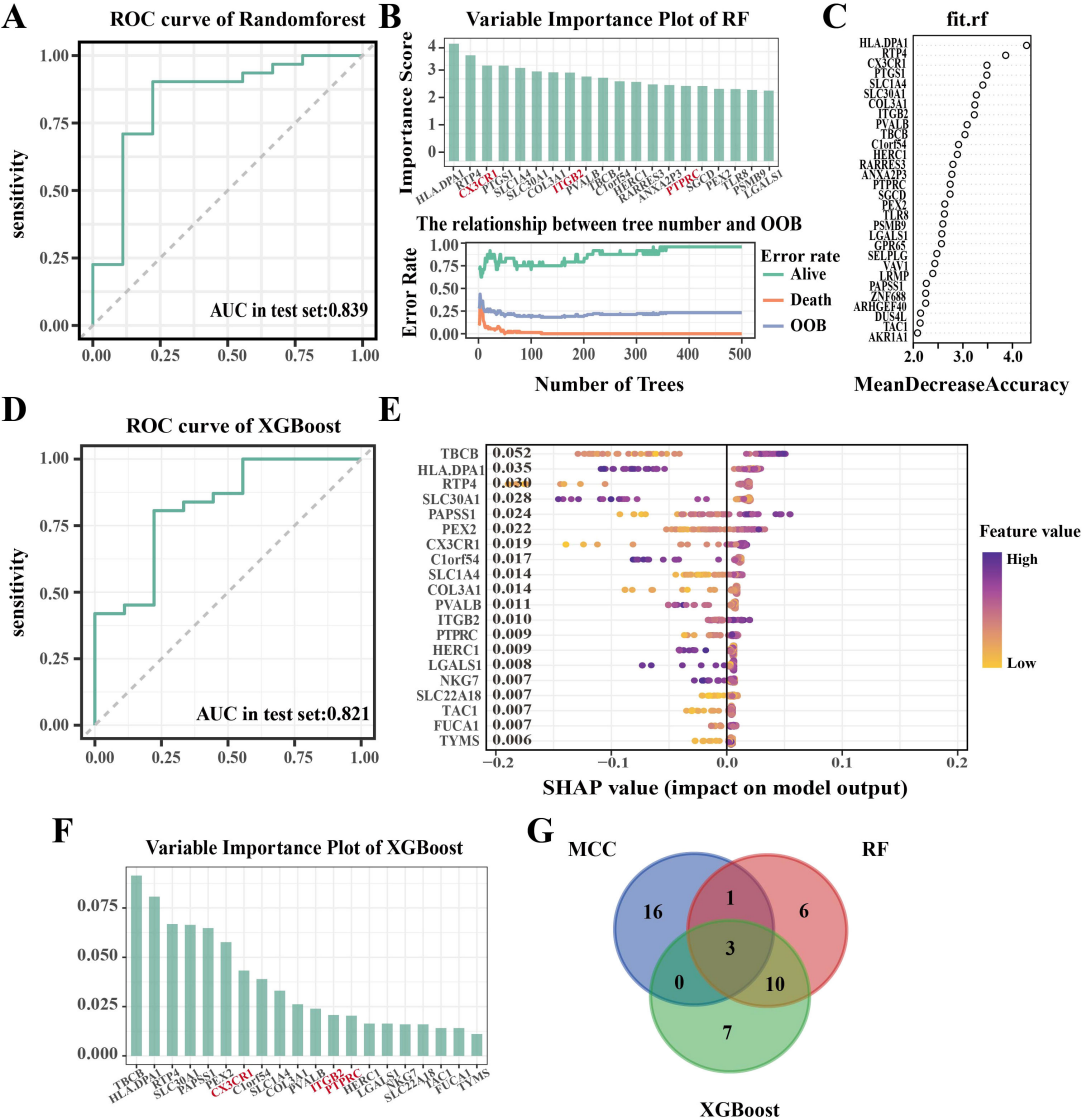
Identification of Hub Genes Using ML. **(A)** ROC curve of RF model. **(B, C)** Top 20 genes ranked by the RF model; **(D)** ROC curve of the XGBoost model; **(E, F)** Top 20 genes ranked by the XGBoost model; **(G)** Intersection of MCC, RF, and XGBoost results to identify hub genes.

PPI analysis of 533 OA-associated genes was performed using the STRING database. The CytoHubba plugin was employed to rank nodes using the MCC method, identifying the top 20 genes (**Figures 5A, B**). The MCODE scores for each gene are summarized in **Supplementary Table 4**. To further identify core genes associated with OA, two ML methods (XGBoost and RF) were applied to the 533 intersection genes. The Random Forest method identified 20 hub genes (**Figures 6A–C**), while the XGBoost method identified another 20 hub genes (**Figures 6D–F**). By comparing the results from these two methods with the 20 genes identified from the PPI network, three common genes—PTPRC, ITGB2, and CX3CR1—were identified and selected as the final hub genes associated with OA (**Figure 6G**).

To assess the universal applicability of the model’s screening features, we employed the independent dataset GSE46750 as a validation set. Model performance was evaluated by calculating the AUC and comparing feature scores between the OA group and control group within the dataset. Results demonstrated successful validation of the model on the independent dataset. The model’s AUC value was significantly >0.5, indicating the robustness and universality of these features across different datasets. Furthermore, the feature scores in the OA group were significantly higher than those in the control group (p < 0.05), indicating that this gene feature is biologically relevant and associated with the pathogenesis of OA (**Supplementary Figures 2A–D**).

To reinforce the model’s biological robustness, we introduced a new dataset (GSE98918). After performing differential analysis on this dataset, we constructed the RF model using the same method. and screened the top 20 genes by feature value. The results indicate that the 20 genes identified through screening overlap with previously identified genes, and CX3CR1, PTPRC, and ITGB2 were again demonstrated to exhibit significant features across different samples, further validating biological robustness (**Supplementary Figure 2E**).

### 3.5 Validation of hub genes

**Figure 7:**
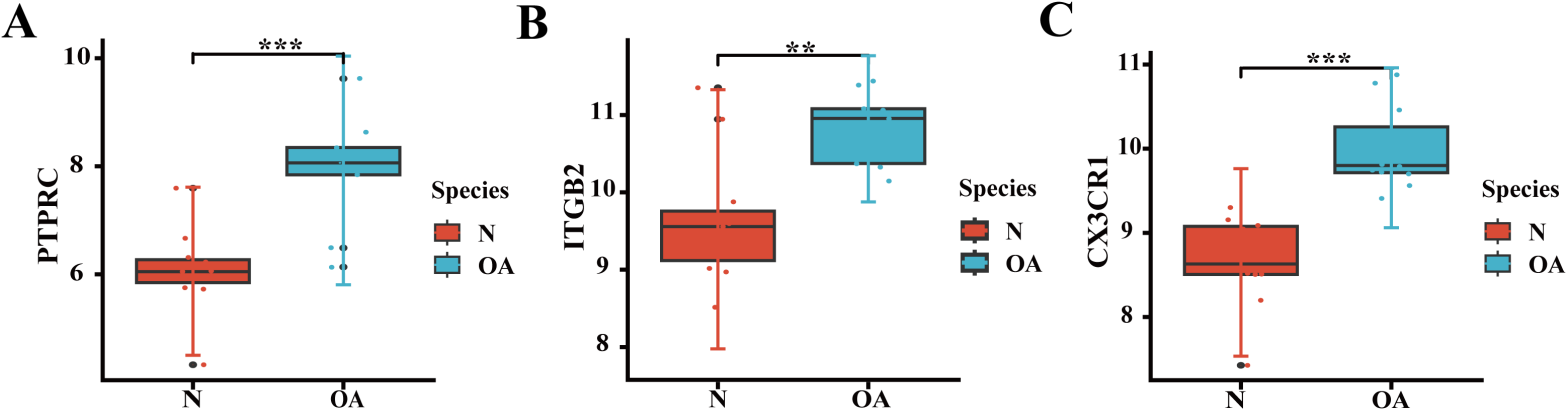
Validation of hub genes. **(A-C)** Boxplot showing the expression differences of PTPRC, ITGB2, CX3CR1between OA and normal samples. **p < 0.01, ***p < 0.001.

To validate the hub genes, the validation dataset GSE98918 was first used to investigate whether the expression levels of the core genes differed between OA and normal samples. The findings indicated that all three hub genes were significantly differentially expressed (**Figures 7A–C**).

**Figure 8:**
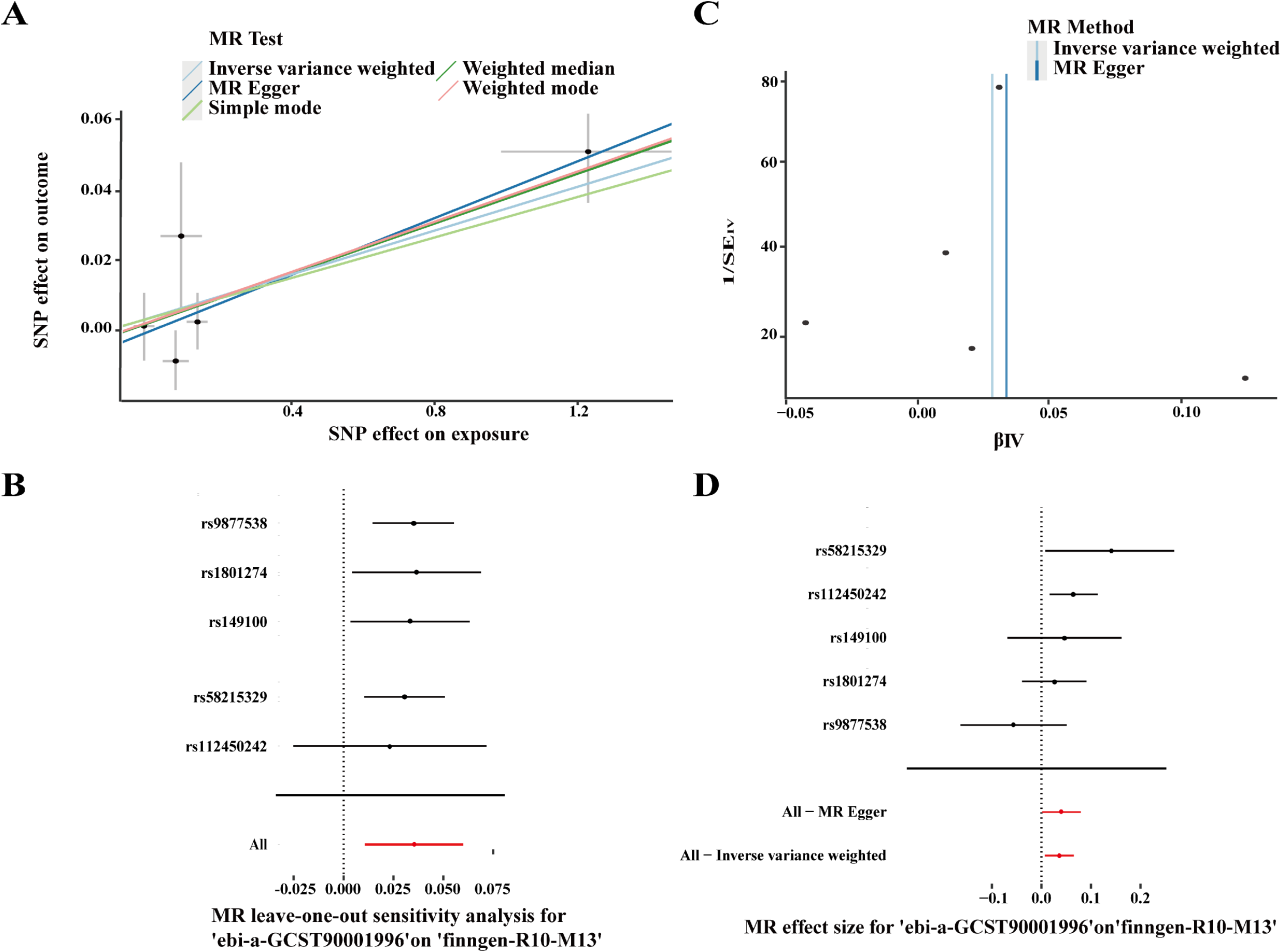
MR Results for CX3CR1. **(A)** Scatter plot of causal SNP effects for CX3CR1 and OA; **(B)** Forest plot of leave-one-out sensitivity illustrating the causal effects of CX3CR1-associated SNPs on OA; **(C)** Funnel plot for causal SNP effects of CX3CR1 and OA; **(D)** MR Egger regression intercept plot for causal SNP effects of CX3CR1 and OA.

MR analysis can directly reveal causal relationships between exposure and outcomes. To further validate the hub genes, MR analysis was conducted to investigate the causal relationship between the hub genes and OA. The IVW approach demonstrated a significant association (OR = 1.032, 10% CI = 1.007–1.057, P = 0.012), which was further supported by the weighted median (OR = 1.033, 56% CI = 1.006–1.062, P = 0.0178) and weighted mode methods (OR = 1.035, 51% CI = 1.009–1.062, P = 0.043). These findings suggest a positive association between CX3CR1 and OA. A scatter plot (**Figure 8A**) illustrates the effect sizes of CX3CR1 and OA SNPs. After sequentially removing each SNP, systematic association analyses were conducted on the remaining SNPs, yielding consistent results that indicate all SNPs exhibit significant causal relationships. This also confirmed that no dominant SNPs were driving the association between CX3CR1 levels and OA and that the previous MR results were valid (**Figure 8B**), verifying the robustness of the analysis. Cochran’s Q test indicated no heterogeneity among instrumental variables (IVs) (P > 0.05) (**Figure 8C**), confirming the absence of significant heterogeneity among SNPs. Furthermore, the intercept value in MR-Egger’s regression analysis did not indicate horizontal pleiotropy (P > 0.05) (**Figure 8D**), confirming that pleiotropy did not bias causal effects and demonstrating the robustness of MR results. Although the MR estimates in our study yield relatively small odds ratios (ORs ≈ 1.03), this is consistent with the genetic architecture of many complex diseases. Genome−wide association studies have shown that individual common genetic variants typically confer only modest increases in disease risk, reflecting a polygenic model in which many small−effect loci contribute to overall susceptibility (37). Additionally, theoretical and empirical studies indicate that genetic effect sizes for complex traits can be underestimated when analyzed by single−variant methods, especially in the presence of many interacting loci (38). Therefore, even small effect sizes observed in MR analyses can be biologically meaningful when interpreted in the context of polygenic disease architecture.

**Figure 9:**
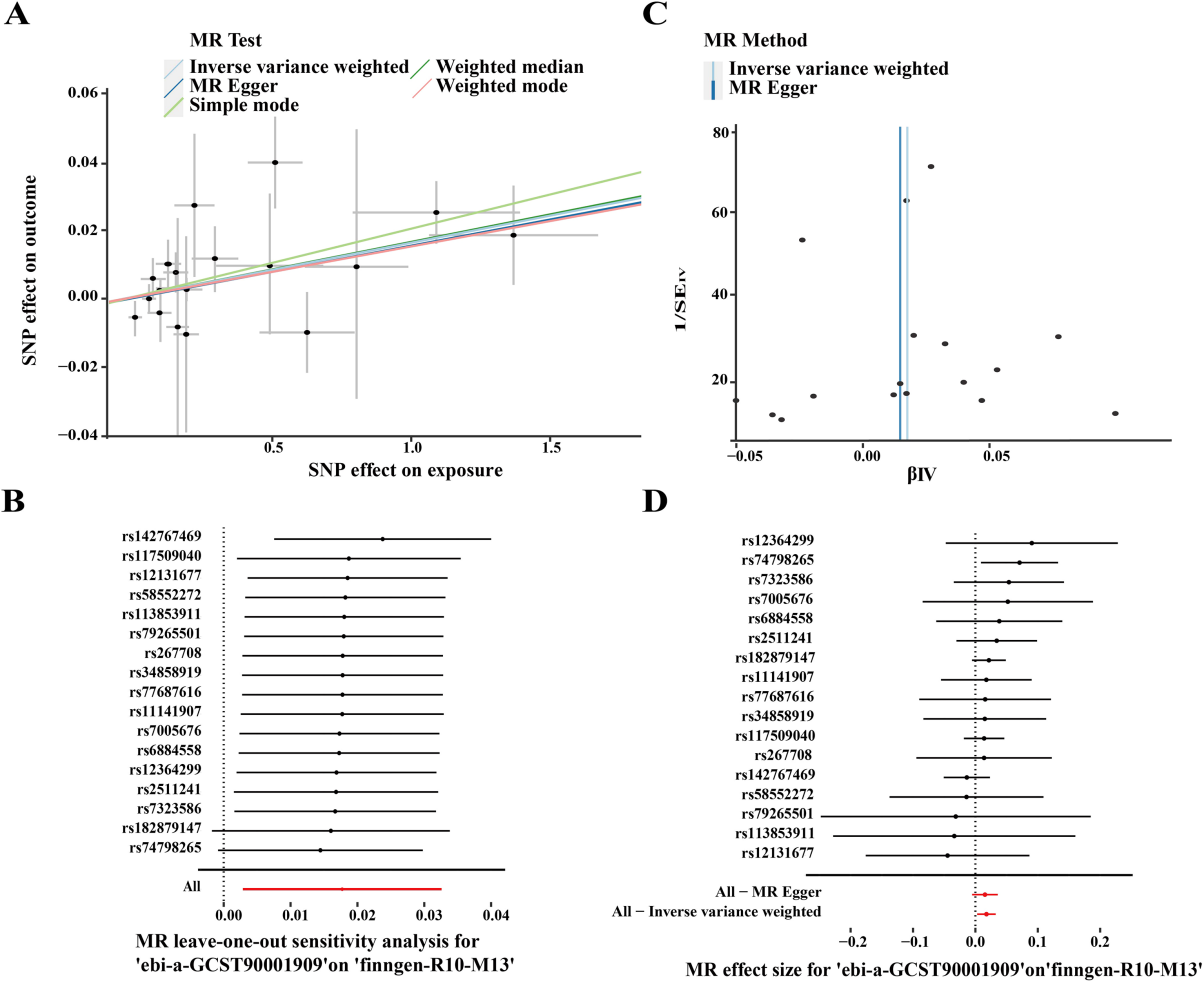
MR Results for PTPRC. **(A)** Scatter plot of causal SNP effects for PTPRC and OA; **(B)** Forest plot of leave-one-out sensitivity illustrating the causal effects of CX3CR1-associated SNPs on OA; **(C)** Funnel plot for causal SNP effects of PTPRC and OA; **(D)** MR Egger regression intercept plot for causal SNP effects of PTPRC and OA.

To investigate the association between PTPRC (exposure) and OA (outcome), seventeen SNPs were selected. The results revealed significant associations using the IVW method (OR = 1.017, 10% CI = 1.002–1.033, p = 0.019). These findings suggest a positive association between PTPRC and OA. A scatter plot (**Figure 9A**) illustrates the effect sizes of PTPRC and OA SNPs. After sequentially removing each SNP, systematic association analyses were conducted on the remaining SNPs, yielding consistent results that indicate all SNPs exhibit significant causal relationships. This also confirmed that no dominant SNPs were driving the association between PTPRC levels and OA and that the previous MR results were valid (**Figure 9B**), verifying the robustness of the analysis. Heterogeneity analysis using Cochran’s Q test showed no significant heterogeneity among the instrumental variables (P > 0.05) (**Figure 9C**). Furthermore, the intercept value in MR-Egger’s regression analysis did not indicate horizontal pleiotropy (P > 0.05) (**Figure 9D**), confirming that pleiotropy did not bias causal effects and demonstrating the robustness of MR results. All results are summarized in **Supplementary Table 5**.

To validate the biological significance of the target genes, we performed WB analysis for PTPRC, ITGB2, and CX3CR1. In the protein expression analysis, we found that PTPRC, ITGB2, and CX3CR1 were significantly more highly expressed in the OA group than in the NC group (**Supplementary Figure 6**). This validated their accuracy as OA target genes.

### 3.6 Immune infiltration

**Figure 10:**
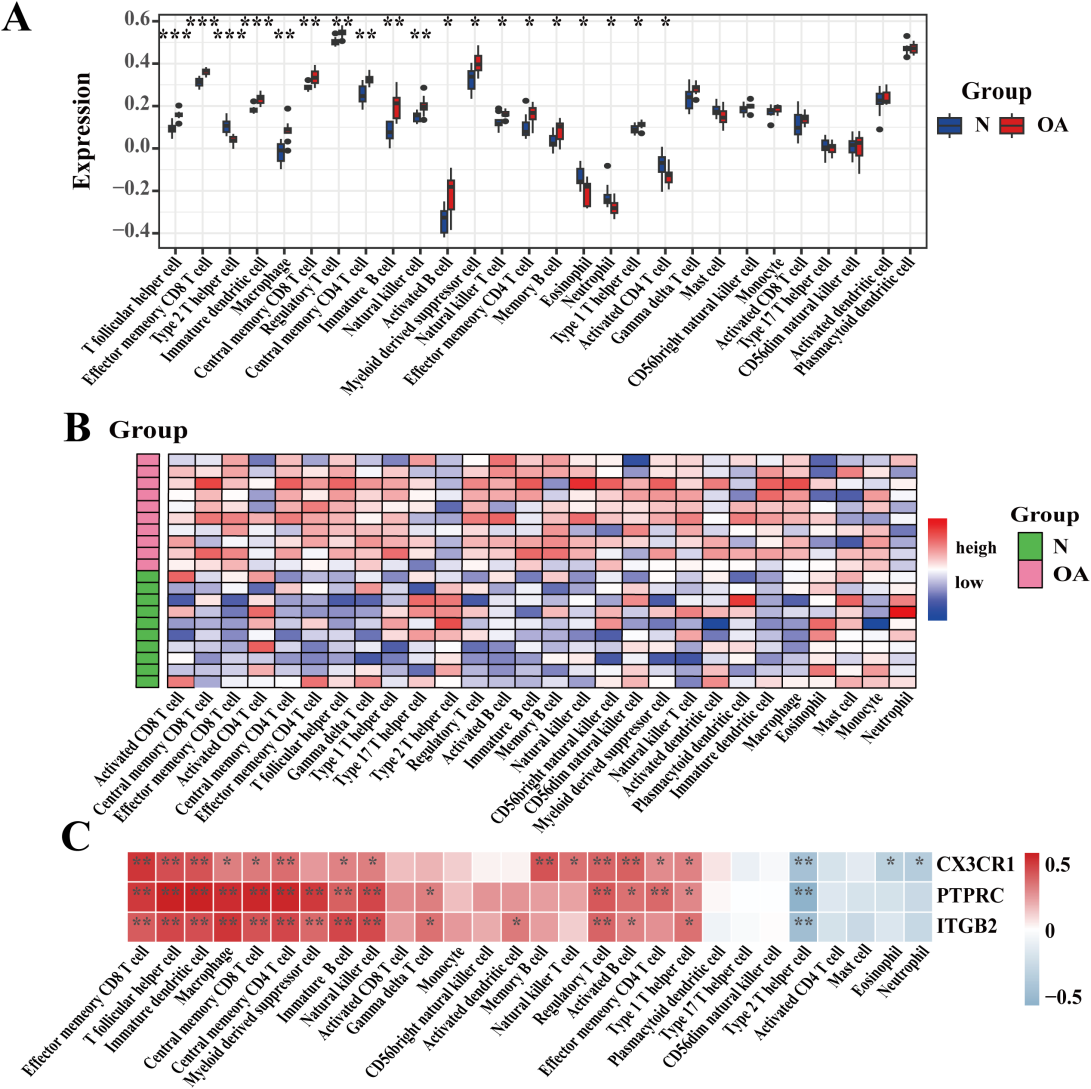
Immune cell infiltration levels in the OA and normal control groups **(A)** Boxplot of infiltration levels; **(B)** Heatmap of infiltration levels; **(C)** Infiltration levels of the three hub genes in immune cells. *p < 0.05, **p < 0.01, ***p < 0.001.

The ‘GSVA’ software package was used to analyze immune cell infiltration patterns, examining the distribution of immune cell types between OA and healthy groups and evaluating the association between key genes and immune cells. A significant growth in the infiltration of B cells, macrophages, and NK cells was observed in the OA group relative to controls (**Figures 10A, B**). Additionally, myeloid-derived suppressor cells (MDSCs) exhibited an upward trend, while eosinophil and neutrophil infiltration levels were slightly decreased. These findings suggest that the hub genes PTPRC, CX3CR1, and ITGB2 may contribute to the onset and progression of OA by influencing immune cells such as B cells, NK cells, and macrophages. Furthermore, a strong correlation was observed between the hub genes and immune cells, indicating that these hub genes might promote OA development and progression through their effects on NK cells and other immune cells (**Figure 10C**). It should be noted that cartilage tissue contains relatively low numbers of immune cells. Therefore, immune infiltration results derived from bulk transcriptomic data should be interpreted with caution. These signals may reflect immune-related transcriptional signatures rather than direct measurements of immune cell abundance. The association between key genes and immune cell populations reflects immune-related transcriptional signatures, which may involve the contribution of immune cells, but these results should not be directly interpreted as quantifying immune cell infiltration.

### 3.7 GSEA analysis of hub genes

To further explore the potential mechanisms by which hub genes influence the development and improvement of OA, single-gene GSEA analysis was conducted. For the KEGG gene sets, genes highly correlated with PTPRC were enriched in B CR signaling, cell adhesion molecules, and primary immunodeficiency diseases. Genes highly correlated with CX3CR1 were enriched in pathways such as oxidative phosphorylation, spliceosome, JAK-STAT signaling, and lysosome. Similarly, genes highly correlated with ITGB2 were enriched in primary immunodeficiency, oxidative phosphorylation, chemokine signaling, and lysosome. These findings suggest that these three core genes may act synergistically across multiple pathways to influence the progression of OA (**Supplementary Figure 3**).

### 3.8 Single-cell sequencing

**Figure 11:**
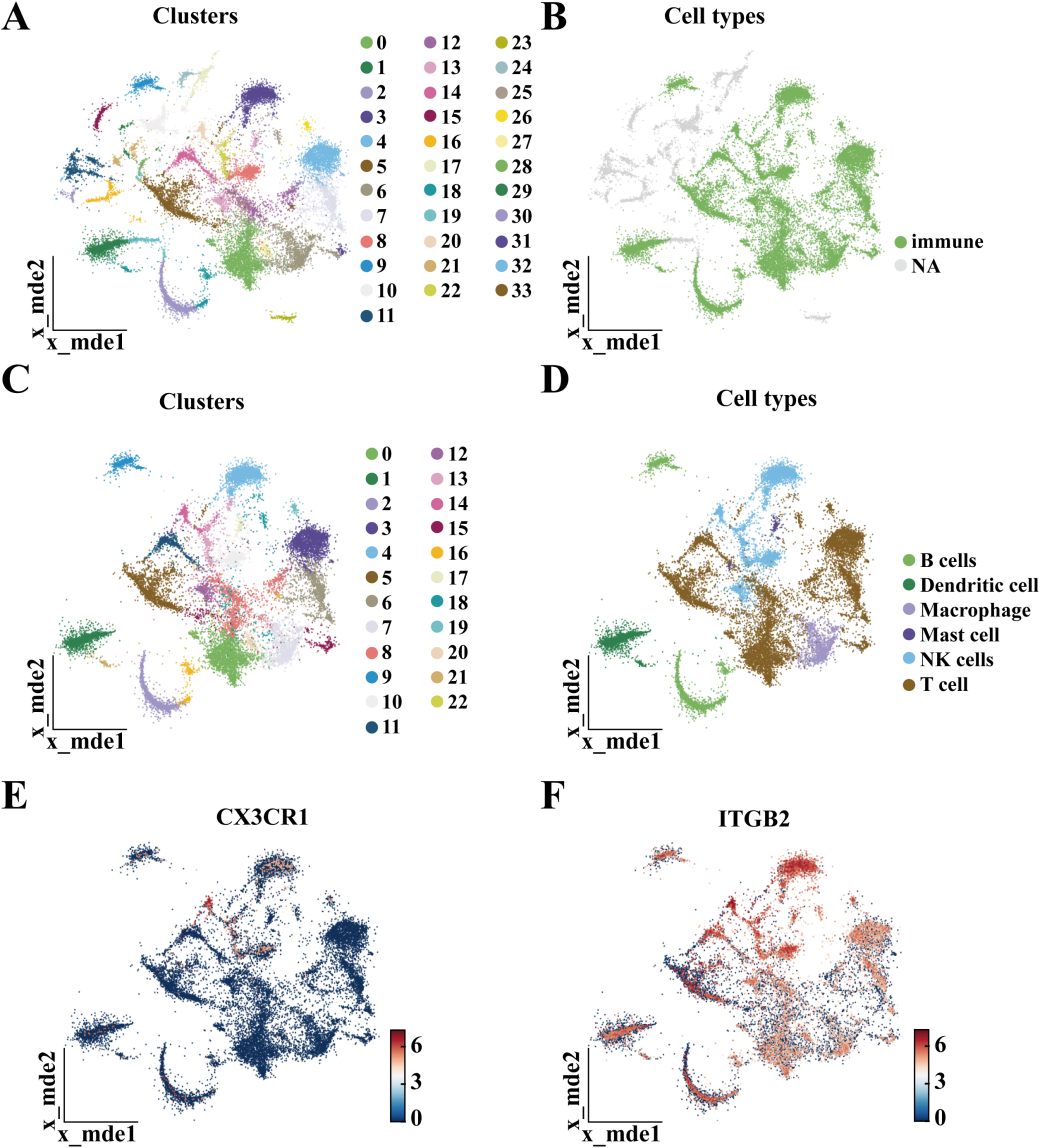
Single-cell sequencing of chondrocytes in four OA patients. **(A)** First clustering of chondrocytes into 33 clusters; **(B)** Clustering results after the first annotation; **(C)** Second clustering of chondrocytes into 22 clusters; **(D)** Clustering results after the second annotation; **(E)** UMAP distribution of CX3CR1 expression; **(F)** UMAP distribution of ITGB2 expression.

In the GSE196678 dataset, clustering was performed at resolution = 1, yielding 33 clusters (**Figure 11A**). Using CD45 as a biomarker, single-cell data were categorized into immune and non-immune cell groups (**Figure 11B**). The immune cell group was then reclustered at resolution = 1, resulting in 22 clusters (**Figure 11C**). Using established marker genes, the identified clusters were assigned to specific cell types and categorized into six principal subpopulations, each characterized by distinct marker gene profiles (**Figure 11D**). UMAP plots illustrated the distribution of hub gene expression among different cellular subpopulations. (**Figures 11E, F**), revealing that hub genes may influence the onset and progression of OA through their effects on immune cells such as B cells, macrophages and NK cells.

In the GSE152805 dataset, data from three diseased medial cartilage samples and three relatively unaffected lateral tibial cartilage samples were integrated, its batch effects were removed using Harmony. Clustering at resolution = 1 resulted in 20 clusters, which were identified as different cell types based on cartilage-related single-cell marker genes. These cells were classified into 10 major subgroups, each exhibiting a unique set of characteristic marker genes. These subgroups include:EC cluster (marked by CHRDL2, FRZB, and CYTL1);RegC cluster (marked by CHI3L1 and CHI3L2);RepC cluster (marked by CILP2, CILP, and OGN);HomC cluster (marked by HSPA1B, HSPA1A, HSPA6, DDIT3, and JUN); Pre hypertrophic chondrocyte (preHTC) cluster (marked by PRG4, ABI3BP, and CRTAC1);Hypertrophic chondrocyte (HTC) cluster (marked by SPP1, IBSP, and COL10A1);Pre-fibrocartilage (preFC) cluster (marked by COL27A1, PLCG2, and WWP2);Fibrocartilage (FC) cluster (marked by MMP2, COL1A1, and COL1A2);Pre-inflammatory chondrocyte (preInfC) cluster (marked by IFI16 and IFI27);Inflammatory chondrocyte (InfC) cluster (marked by CXCL8, CD74, and GPR183) (39). UMAP plots revealed the distribution of different cell types in OA and relatively unaffected samples, showing that OA samples were primarily enriched in reparative chondrocytes, pre hypertrophic chondrocyte, hypertrophic chondrocytes, inflammatory chondrocytes, and pre-inflammatory chondrocytes.Using the Python-based CellPhoneDB library, intercellular interaction levels among different subpopulations were estimated, predicting cell-cell interactions in scRNA-seq data. This provides insights for further research into the roles of different cell subpopulations in OA progression (**Supplementary Figure 4**).

### 3.9 PTPRC is essential for chondrocyte damage

Preliminary experimental results from our research group indicate that Src and FAK are strongly associated with bone diseases, playing a crucial role in bone metabolism by regulating the balance between bone resorption and formation. KEGG analysis suggests that BCR signaling pathway may serve as a potential pathway for pivotal genes. Literature review revealed Src protein inhibitors demonstrated efficacy in alleviating OA (40). Concurrently, PTPRC plays a key role in BCR signaling by activating Src family kinases, and the BCR signaling pathway activates the NF-κB signaling pathway through multiple molecules and mechanisms (41). To elucidate this specific signaling pathway, PTPRC was selected for validation.

**Figure 12:**
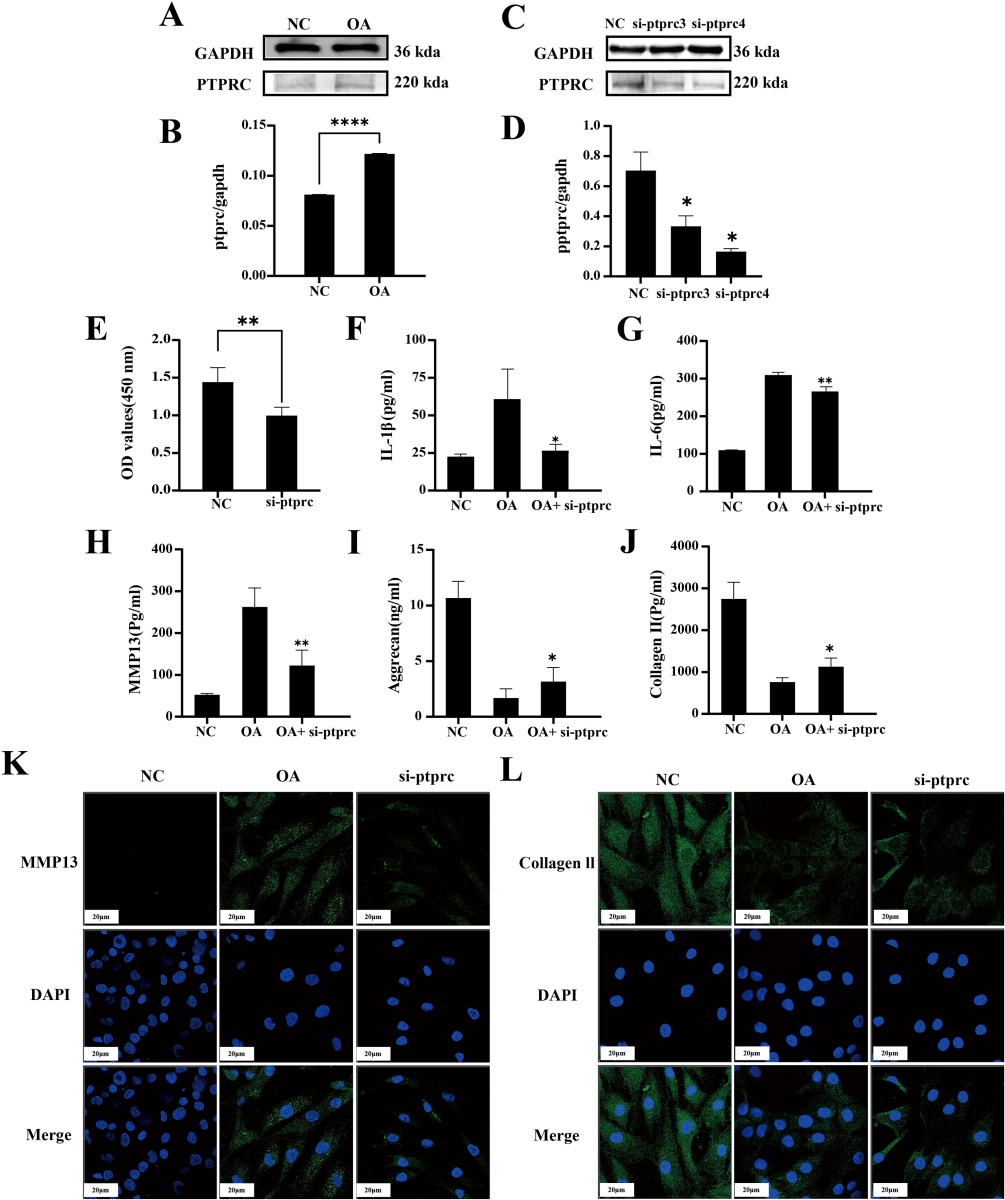
PTPRC is essential for chondrocyte damage. **(A)** WB analysis of PTPRC ex-pression in normal chondrocytes and inflammatory chondrocytes, and **(B)** corresponding semi-quantitative analysis; **(C)** WB analysis of PTPRC expression after siRNA treatment for 48 h, and **(D)** corresponding semi-quantitative analysis; **(E)** Proliferation of inflammatory chondrocytes following si-PTPRC treatment for 48 h; **(F)** ELISA analysis results for IL-1β secreted by chondrocytes; **(G)** ELISA analysis results for IL-6 secreted by chondrocytes. **(H)** ELISA analysis of MMP13; **(I)** ELISA analysis of aggrecan. **(J)** ELISA analysis of Col2a1; **(K–L)** Immunofluorescence staining results for MMP13 and Col2a1 in different treated cell groups. *p < 0.05, **p < 0.01, ***p < 0.001, ****p < 0.0001, n=3.

Primary chondrocytes were isolated from patients with OA (**Supplementary figure 5**). Detailed clinical information of the OA samples, including age, sex, KL grade, BMI, and WOMAC scores, is provided in **Supplementary Table 1**. To determine whether PTPRC is a target gene in OA, its expression was examined in the isolated chondrocytes. WB analysis revealed that PTPRC protein levels were significantly higher in OA chondrocytes compared to normal controls (**Figures 12A, B**), as quantified by semi-quantitative grayscale values normalized to an internal reference. This confirms that PTPRC is upregulated in OA and supports its candidacy as a hub gene.

During inflammatory states, chondrocytes release inflammatory mediators and matrix metalloproteinases, leading to altered chondrocyte function and subsequent degradation of the cartilage matrix (42, 43). To further explore the functional role of PTPRC in articular chondrocytes, primary chondrocytes were transfected with si-PTPRC, resulting in efficient silencing of PTPRC expression (**Figures 12C, D**). Following knockdown, CCK-8 and ELISA assays showed decreased proliferation of inflammatory chondrocytes and reduced secretion of IL-1β and IL-6 (**Figures 12E–G**). Additionally, expression of ECM-related genes Mmp13, Acan, and Col2a1 was modulated consistent (**Figures 12H–J**). These results were consistent with immunofluorescence findings (**Figures 12K, L**).

These experiments suggest that PTPRC may be involved in OA-related chondrocyte dysfunction, including inflammatory proliferation, cytokine release, and ECM remodeling. All cellular experiments were performed with at least three independent biological replicates (n=3), each replicate comprising multiple technical replicates to ensure reproducibility and statistical reliability of the results.

### 3.10 Simulating PTPRC knockdown to regulate NK Cells in inflammatory environments

PTPRC, as a marker for immune cell subpopulation classification, demonstrates a clear influence on the immune microenvironment. Experimental evidence indicates that PTPRC restricts early NK cell development (44). To further investigate PTPRC’s key regulatory role in the immune microenvironment, we performed virtual knockdown of PTPRC. Based on the single-cell transcriptomics dataset, we employed IL-35 induction to simulate NK cells under chronic inflammation conditions (45). Results indicate that PTPRC knockdown not only disrupts gene expression associated with immune responses but also significantly impacts the expression network of ribosome-related genes (**Supplementary Figure 7**) (**Supplementary Table 1**). This suggests that PTPRC deficiency may reshape the immune microenvironment and interfere with inflammatory response mechanisms. GPM6A is expressed across multiple cell types, playing a particularly crucial role in the immune system. It participates in regulating the activity and interactions of immune cells, aiding the body in combating infections and diseases (46). Ribosomal proteins (RPL10, RPLP1) exhibit correlations with inflammation regulation and immune function. Recent studies increasingly demonstrate that beyond their classical translational roles, ribosomal proteins possess atypical immunomodulatory functions and participate in inflammatory processes (47). Collectively, this evidence suggests that PTPRC serves not only as a critical immune regulatory node but may also contribute to the maintenance or resolution of chronic inflammatory states through interactions with inflammatory response networks.

## 4 Discussion

In this study, gene expression data from joint tissues in the GEO database were analyzed, identifying DEGs associated with OA. WGCNA revealed the turquoise module to be highly correlated with OA. Further, three hub genes (CX3CR1, PTPRC, and ITGB2) were identified using PPI network construction and ML algorithms (Random Forest and XGBoost). These analyses primarily reflect statistical correlations and biological associations at the level of gene expression, rather than directly proving causality. MR analysis confirmed the positive correlation of these hub genes with OA, emphasizing their potential as diagnostic biomarkers. WB results revealed significantly higher protein expression levels of ITGB2, PTPRC, and CX3CR1 in OA samples compared to NC, thereby supporting the potential role of these genes in OA at the protein expression level. Notably, immune cell infiltration analysis revealed that hub genes exhibit immune-related transcriptional characteristics. Single-cell sequencing analysis highlighted a strong association between hub genes and NK cells, suggesting they may play a pivotal role in immune regulation during the progression of OA. This study extends existing research by demonstrating the central role of genes in OA nosogenesis and revealing their potential value in early detection and targeted therapy. This study also identified a novel target for OA—PTPRC. *In vitro* experiments revealed its impact on chondrocyte repair, and its interaction with the immune microenvironment may offer new insights for OA treatment.

Among the three hub genes identified in this study, PTPRC emerged as a central immunoregulatory node linking immune activation to cartilage degeneration in OA. PTPRC is a leukocyte-specific protein tyrosine phosphatase that plays a critical role in regulating immune cell activation thresholds and signal transduction (48, 49). In this study, PTPRC was significantly upregulated in OA cartilage tissues, with enriched pathways including BCR signaling, cell adhesion molecules, and primary immunodeficiency, highlighting its strong association with immune dysregulation in the osteoarthritic microenvironment. Functionally, both *in vitro* experiments and virtual knockdown analyses support a pivotal role for PTPRC in modulating inflammatory responses and extracellular matrix homeostasis. Knockdown of PTPRC significantly attenuated inflammatory factor release and reduced matrix degradation in chondrocytes, indicating that PTPRC-mediated immune activation contributes directly to cartilage structural damage. Moreover, in silico perturbation of PTPRC in NK cells revealed pronounced alterations in the immune microenvironment, suggesting that PTPRC regulates cartilage degeneration not only through intrinsic chondrocyte responses but also via immune cell–mediated mechanisms. These findings position PTPRC at the interface between immune regulation and tissue integrity, underscoring its relevance as a potential immunomodulatory target for cartilage repair strategies.

In addition to PTPRC, CX3CR1 and ITGB2 were identified as immune-related hub genes that may function cooperatively to shape the inflammatory microenvironment in OA. CX3CR1 is a membrane-associated receptor that mediates immune cell chemotaxis and adhesion through its interaction with fractalkine (CX3CL1), and is a member of the chemokine receptor superfamily (50, 51), while ITGB2 is a β2 integrin subunit involved in leukocyte adhesion, migration, and immune synapse formation. In this study, both genes were significantly upregulated in OA cartilage tissues and exhibited strong associations with NK cell infiltration. Previous studies have shown that CX3CR1-CX3CL1 signaling can enhance inflammatory responses and promote cartilage degradation through increased matrix metalloproteinase production (52–55), while ITGB2 facilitates immune cell recruitment and retention within inflamed tissues (56). Consistent with these observations, our enrichment and immune infiltration analyses suggest that CX3CR1 and ITGB2 contribute to immune cell trafficking and activation in OA, thereby amplifying inflammatory signaling and tissue damage. Rather than acting as isolated drivers, these molecules appear to cooperate with PTPRC to reinforce immune-mediated cartilage degeneration by promoting immune cell infiltration and sustaining inflammatory signaling within the joint microenvironment.

The three hub genes determined in this study—CX3CR1, PTPRC, and ITGB2—are closely interconnected in their roles within immune regulation and inflammation. Functionally, all three proteins are involved in immune cell activation, migration, and signaling, with a shared preference for modulating pathways such as antigen processing, JAK-STAT signaling, and focal adhesion. These proteins also exhibit overlapping contributions to NK cell and B cell activity, which are critical in both OA progression and immune surveillance. Notably, CX3CR1 primarily mediates chemotaxis and inflammatory signaling, while PTPRC regulates leukocyte activation, and ITGB2 facilitates cell adhesion and migration. Together, they form a network that drives immune cell infiltration, inflammation, and tissue remodeling, hallmarks of OA pathology. Beyond OA, these proteins are widely implicated in tumor biology, where they interact with pathways such as focal adhesion and JAK-STAT to promote tumor progression, immune evasion, and metastasis. These findings suggest a broader functional preference for immune regulation and inflammation, providing a foundation for targeted therapies in both OA and related inflammatory or neoplastic diseases.

The pathogenesis of OA is highly complex, involving diverse pathways such as NF-κB, and focal adhesion signaling. Single-gene GSEA analysis revealed that CX3CR1, PTPRC, and ITGB2 were enriched in key immune regulatory pathways and cell communication, including antigen processing, JAK-STAT signaling, and NOD-like receptor signaling. These findings suggest a dynamic interplay between immune responses, inflammation, and cell-cell interactions in OA progression. Previous studies suggest potential downstream targets for these genes, including PTPRC can regulate Pyk2/FAK activity through the activity of Src family kinases (57), the interaction between ITGB2 and STC2 may participate in the regulation of downstream FAK (56), and enhanced FAK and Src phosphorylation by CX3CR1 overexpression (58). It follows that the enrichment of focal adhesion pathways highlights the potential involvement of mechanotransduction in OA, a concept increasingly recognized in recent studies. Moreover, immune-related transcriptional signature analysis indicated relatively higher signals associated with T cells, B cells, NK cells, and macrophages in OA joint tissues compared to normal controls, suggesting an increase in immune-associated activity, while acknowledging that these signals may reflect contributions from both infiltrating immune cells and chondrocyte-intrinsic immune gene expression. These findings suggest that the hub genes may exacerbate OA progression by influencing immune cell infiltration and activation.

This study identified key driver genes (PTPRC, CX3CR1, ITGB2) closely associated with the OA immune microenvironment and revealed that PTPRC plays a central role in regulating local immune responses. PTPRC is highly expressed in immune cells, particularly NK cells, and its knockdown significantly alters immune activity, potentially mitigating the persistent damage to chondrocytes caused by inflammatory signals. Following cartilage injury, the joint microenvironment typically exhibits chronic inflammation, increased pro-inflammatory cytokine release, and abnormal immune cell recruitment, which accelerates cartilage degradation. Our findings highlight that modulating the local immune microenvironment—especially through the activity of PTPRC—may provide a therapeutic avenue for controlling inflammation and preserving cartilage integrity in OA.

This study has several limitations. First, although three potential hub genes (CX3CR1, PTPRC, and ITGB2) were identified through integrated bioinformatic analyses, the sample size of this study is relatively limited, particularly in the bioinformatics analysis section. Although we enhanced the robustness of our findings by integrating multiple independent cohorts and employing multi-method cross-validation, future research should incorporate larger sample sizes to further validate the expression patterns and functional roles of key genes. Second, the transcriptomic data were derived from public datasets, and potential heterogeneity across cohorts may have influenced gene selection. Future studies using more homogeneous tissue sources or single-cell data will further validate these findings. Third, results from immune cell infiltration profiling and single-cell analysis imply that these genes may influence immune-related processes, warranting additional investigation into their specific mechanisms of action.

## 5 Conclusions

In summary, this study utilized an integrative approach combining bioinformatics and ML to identify and validate three OA-associated hub genes: CX3CR1, PTPRC, and ITGB2. A detailed study of the mechanism of action of PTPRC revealed its crucial role in mitigating the progression of OA. These genes not only serve as potential diagnostic biomarkers but also shed light on the molecular mechanisms underlying OA progression, particularly through their roles in immune regulation and signaling.

## Data Availability

The original contributions presented in the study are included in the article/**Supplementary Material**. Further inquiries can be directed to the corresponding authors.
