# Supplementary material for: Exploring immune modulation in osteoarthritis: identifying key biomarkers and the role of PTPRC in regulating immune microenvironment for therapeutic intervention
Source: Front Immunol. 2026 Apr 13;17:1795075. doi: 10.3389/fimmu.2026.1795075 (PMC13111039; doi:10.3389/fimmu.2026.1795075)
Supplement: Supplementary file 2 [file Table2.docx]

Supplementary Materials

**The file includes**

Materials and Methods

Figure S1. Intersection Genes and Their Enrichment Analysis.

Figure S2. Independent validation of machine learning algorithms.

Figure S3. Single-Gene Set Enrichment Analysis of Hub Genes.

Figure S4. Single-Cell Sequencing of Chondrocytes in Three OA Patients and Three Normal Samples.

Figure S5. Identification of chondrocytes.

Figure S6. Western blot validation of PTPRC, CX3CR1, and ITGB2 protein expression in OA and NC samples.

Figure S7. Genetic disruption caused by PTPRC knockout in NK cells.

**Materials and methods**

**Acquisition of Microarray Expression Data**

The inclusion criteria for datasets to identify differential genes were as follows: (1) the experimental type was expression profiling by array; (2) the organism was Homo sapiens; (3) datasets were derived from the same platform to enable subsequent batch effect removal. The GSE55235 dataset included 10 OA samples and 10 normal samples; GSE55457 contained 10 OA samples and 10 normal samples; and GSE169077 comprised 6 OA samples and 5 normal samples.

**Data Preprocessing**

Use the “GEOquery” package to extract clinical information and expression matrices from GEO datasets, while converting probe matrices to gene matrices via probe annotation files. When multiple probes mapped to a single gene, calculate their average to represent gene expression. To minimize batch-related bias, the ComBat function within the “sva” package was employed across the three datasets,this preprocessing ensures comparability and minimizes technical variability across datasets.

**Differential Expression and WGCNA were Conducted to Identify Genes of Interest**

Limma package threshold for differential analysis: p < 0.05, |log2 change value| > 0.5

WGCNA standards: Soft threshold power = 12, Minimum module size = 30, Merge cut-off height = 0.25. The correlation between modules and phenotypes was calculated to identify OA-associated modules. Gene significance (GS) and module membership (MM) were used to characterize gene–trait correlations across the selected co-expression modules.

**Selection of Genes Using ML Algorithms**

RF, a bagging ensemble method, utilizes multiple decision trees for classification and regression, while XGBoost, a boosting ensemble method, is a tree-based algorithm. Initial expression data of intersecting genes were preprocessed by adding a "Grade" column, randomly sampled from letters A to F, and one-hot encoded. Data were split into training and testing sets in a 7:3 ratio. The "randomforest" package was used to construct an RF model, with 10-fold cross-validation and ROC curve evaluation to ensure reliability. Feature importance scores were used to rank and select the top 20 genes. Similarly, the "XGBoost" package was used to build an XGBoost model, with the best iteration number determined by 10-fold cross-validation. SHAP values were used to visualize the importance of each feature, and the top 20 genes were selected. To assess the generalizability of the identified 20-gene signature, we used independent dataset (GSE46750), a completely independent validation set that was not involved in feature selection or model training. The model’s performance was evaluated by calculating the AUC and comparing the signature scores between the OA and Control groups in dataset. To determine the biological stability of this model, we also applied the aforementioned method to a new independent dataset (GSE98918).

**MR Analysis**

GWAS data of hub genes (<https://www.ebi.ac.uk/gwas/>) were used as exposure, and OA data from the FinnGen consortium^[1]^ were used as outcomes .In R,OA-associated genes were identified. SNPs associated with exposure factors (P < 1×10^-5) were selected. (Due to an insufficient number of valid instrumental variables (IVs) at a significance threshold of 5×10^-8, a relaxed threshold of 1×10^-5 was adopted, supplemented with sensitivity analysis and robustness tests to strengthen the evidence). Thresholds of r^2 < 0.001 and kb = 10,000 were set to evaluate linkage disequilibrium (LD) among the included SNPs. From the GWAS dataset, β coefficients, P-values, and standard errors for each allele of the identified SNPs were extracted. IVs significantly associated with outcome phenotypes (P < 5×10^-8) were excluded. Next, harmonization was performed by aligning allele directions between exposure-SNPs and outcome-SNPs, removing ambiguous palindromic sequences and incompatible SNPs to enhance the stability of the analysis. A two-sample MR analysis was conducted using five methods: inverse variance weighted (IVW), weighted median, simple mode, weighted mode, and MR-Egger. Horizontal pleiotropy was assessed using the MR-Egger intercept; P ≥ 0.05 indicated the absence of horizontal pleiotropy^[2]^. Cochran’s Q statistic and its corresponding P-value were calculated to determine heterogeneity, with P ≥ 0.05 indicating no significant heterogeneity ^[3]^ . Finally, sensitivity analysis was conducted using the leave-one-out method^[4]^ to evaluate the reliability of the results and the potential influence of individual SNPs.

**Western Blot Analysis**

After extracting total cellular protein, proteins were separated by SDS-PAGE in a 10% gel and transferred to a PVDF membrane. After blocking with 5% BSA for 2 h at room temperature, the membranes were incubated overnight at 4 °C with primary antibodies against GAPDH (1:10,000, 81640-5-RR, Proteintech, USA) and PTPRC (1:5,000, 84405-6-RR, Proteintech, USA), CX3CR1(1:3000, 29819-1-AP, Proteintech, USA), ITGB2(1: 2000, 10554-1-AP, Proteintech, USA). Next day, after three washes with TBST, the membranes were incubated with corresponding secondary antibodies for 1 h at room temperature, followed by visualization using enhanced chemiluminescence (ECL), visualized using a gel imaging system, and the protein bands were analyzed with ImageJ software.

**Gene Set Enrichment Analysis (GSEA)**

A core gene list was extracted from the processed expression matrix in the initial step to construct the core gene expression matrix. Correlation analysis between hub genes and dataset genes was conducted to identify datasets highly correlated with the hub genes, which were then used for enrichment analysis.

**Single-Cell Sequencing Analysis**

Preprocessing was conducted using the "omicverse"^[5]^ and "scanpy" ^[6]^ packages in Python. Quality control thresholds were set as mitoperc = 0.15, nUMIs = 500, and detected genes = 250 to filter low-quality and doublet cells. Gene expression was normalized using the Pearson method, and 3,000 highly variable genes were selected for dimensionality reduction. Cell clusters were identified and annotated using the Leiden algorithm.

**Solation and Culture of Primary Human Chondrocytes**

Articular cartilage specimens obtained from patients undergoing knee arthroplasty were placed in PBS containing 10% penicillin–streptomycin. The cartilage was cut into small pieces using sterile surgical instruments and further minced into fine fragments of approximately 1 mm³ with ophthalmic curved scissors, followed by washing three times with PBS. Yellow, uneven cartilage from necrotic areas on the joint surface was defined as inflammatory cartilage, whereas smooth and morphologically normal tissue surrounding the necrotic regions was defined as normal cartilage. The tissue was first digested with 0.25% trypsin–ethylenediaminetetraacetic acid (EDTA) for 30 min, followed by digestion with 0.2% type II collagenase prepared in DMEM/F12 medium for 24 h. The digested tissue was spun at 50 × g for 5 min, the supernatant was collected and further centrifuged at 300 × g for 5 min. A milky white pellet observed at the bottom of the tube represented the isolated articular chondrocytes. Following removal of the supernatant, chondrocytes were maintained in DMEM/F12 medium containing 10% fetal bovine serum (FBS) and 1% penicillin–streptomycin (PS) at 37 °C in a humidified incubator with 5% CO₂. When cell confluence reached approximately 90%, chondrocytes were passaged, with no more than three passages performed. For all passaging procedures, chondrocytes were detached using 1 mL trypsin–EDTA and collected by centrifugation at 100 × g for 5 min. Chondrocytes at passage three or earlier were used for subsequent experiments.

**Cell Viability Assay**

After enzymatic digestion, chondrocytes were seeded into 96-well plate at a density of 8,000 cells per well, with three replicate wells for each group. Cells were transfected with si-PTPRC for 48 h. After washing with PBS, cells were treated with 200 μL of CCK-8 solution (1:9 dilution in DMEM/F12) and incubated at 37 °C for 2 h, after which optical density was measured at 450 nm.

**Toluidine Blue Histological Staining**

By binding to anionic glycoconjugates such as proteoglycans and glycosaminoglycans, toluidine blue enables the visualization of cartilage matrix components. Chondrocytes were seeded on coverslips in 24-well plates at a density of 20,000 cells per well. Once cells reached approximately 70% confluence, they were fixed in 4% paraformaldehyde for 15 min, followed by staining with 1% toluidine blue for 30 min. Coverslips were rinsed with PBS, mounted on glass slides, and subsequently visualized by light microscopy.

**Cell immunofluorescence staining**

After processing cells according to specified protocols, fix them with paraformaldehyde. Then permeabilize with 0.2% Triton X-100 (20 min) and block with 1% BSA (30 min). Next, cells were incubated overnight at 4°C with primary antibodies: rabbit anti-collagen II antibody (1:200, 28459-1-AP, Proteintech, USA) and rabbit anti-MMP13 antibody (1:200, 83188-2-RR, Proteintech, USA). Following overnight incubation, cells were exposed to fluorescently conjugated goat anti-rabbit IgG (1:500; SA00013-2, Proteintech, USA) for 1 h and then stained with DAPI for nuclear visualization. Fluorescence signals were visualized by microscopy and processed using ImageJ software.

[1] Kurki M I, Karjalainen J, Palta P, et al. FinnGen provides genetic insights from a well-phenotyped isolated population[J]. Nature, 2023, 613(7944): 508-518.

[2] Burgess S, Thompson S G. Interpreting findings from Mendelian randomization using the MR-Egger method[J]. Eur J Epidemiol, 2017, 32(5): 377-389.

[3] Bowden J, Tierney J F, Copas A J, et al. Quantifying, displaying and accounting for heterogeneity in the meta-analysis of RCTs using standard and generalised Q statistics[J]. BMC Med Res Methodol, 2011, 11: 41.

[4] Higgins J P, Thompson S G. Quantifying heterogeneity in a meta-analysis[J]. Stat Med, 2002, 21(11): 1539-1558.

[5] Zeng Z, Ma Y, Hu L, et al. OmicVerse: a framework for bridging and deepening insights across bulk and single-cell sequencing[J]. Nat Commun, 2024, 15(1): 5983.

[6] Wolf F A, Angerer P, Theis F J. SCANPY: large-scale single-cell gene expression data analysis[J]. Genome Biol, 2018, 19(1): 15.


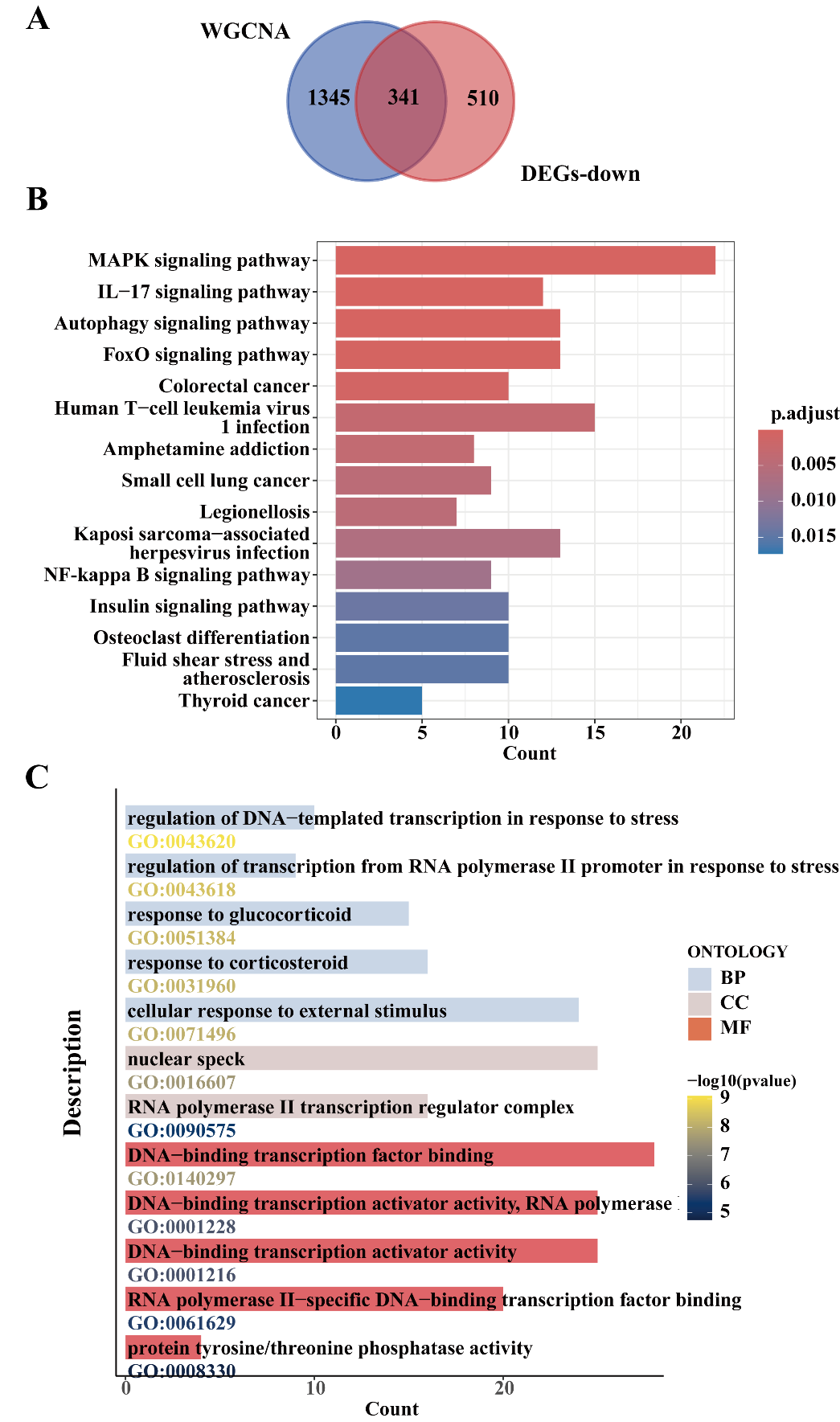


**Figure S1. Intersection Genes and Their Enrichment Analysis.** (A) Intersection genes derived from Downregulated DEGs and co-expressed genes; (B) KEGG pathway analysis of the intersection genes; (C) GO pathway analysis of the intersection genes.


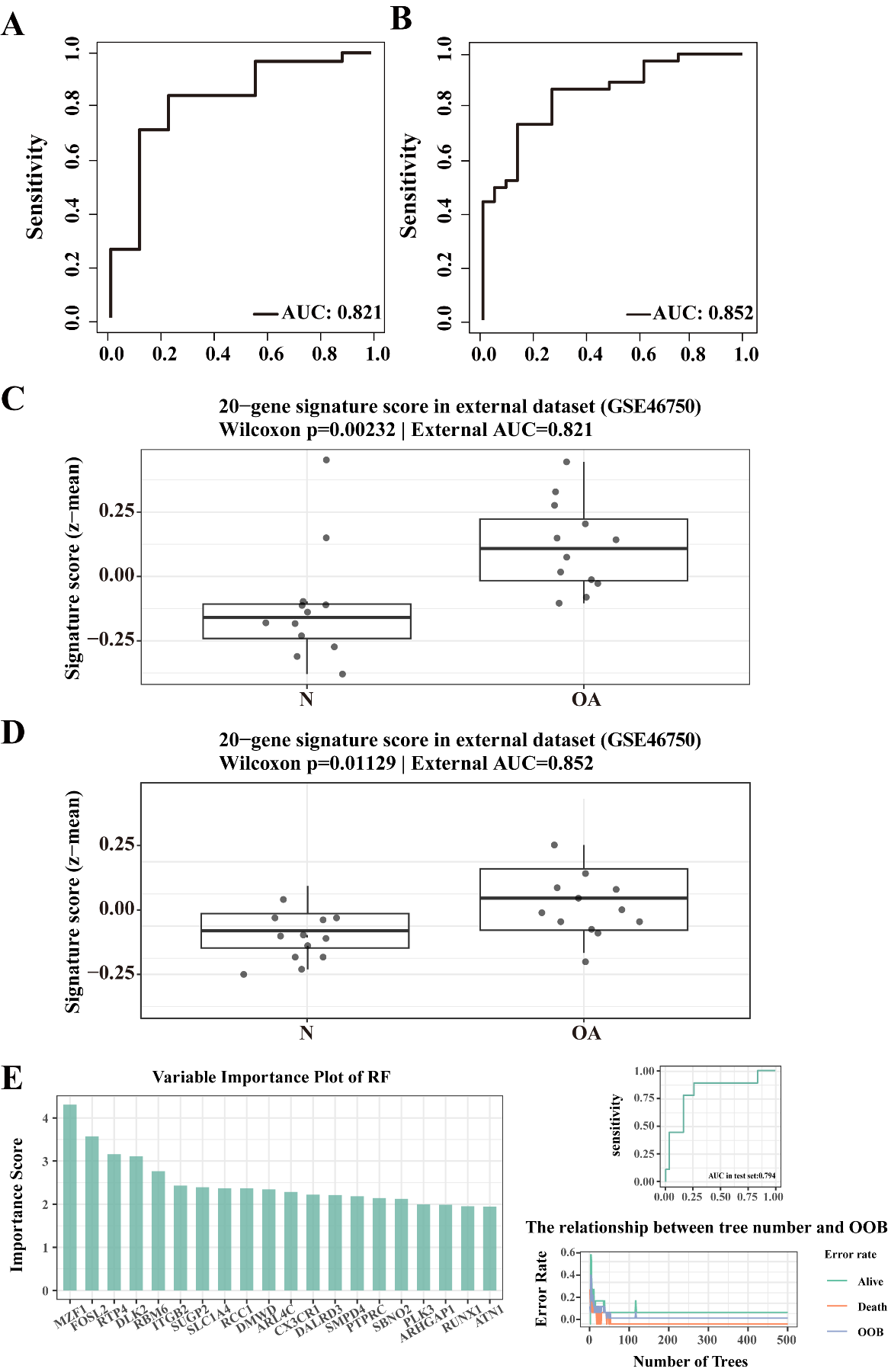


**Figure S2. Independent validation of machine learning algorithms.** (A) Independent validation of the RF method model in GSE46750; (B) Independent validation of the XGBoost method model in GSE46750; (C) Differential expression of the 20 genes selected by RF across different samples in the GSE46750 dataset; (D) Differential expression of the 20 genes selected by XGBoost across different samples in the GSE46750 dataset; (E) 20 key genes identified by the RF model in t GSE98919.


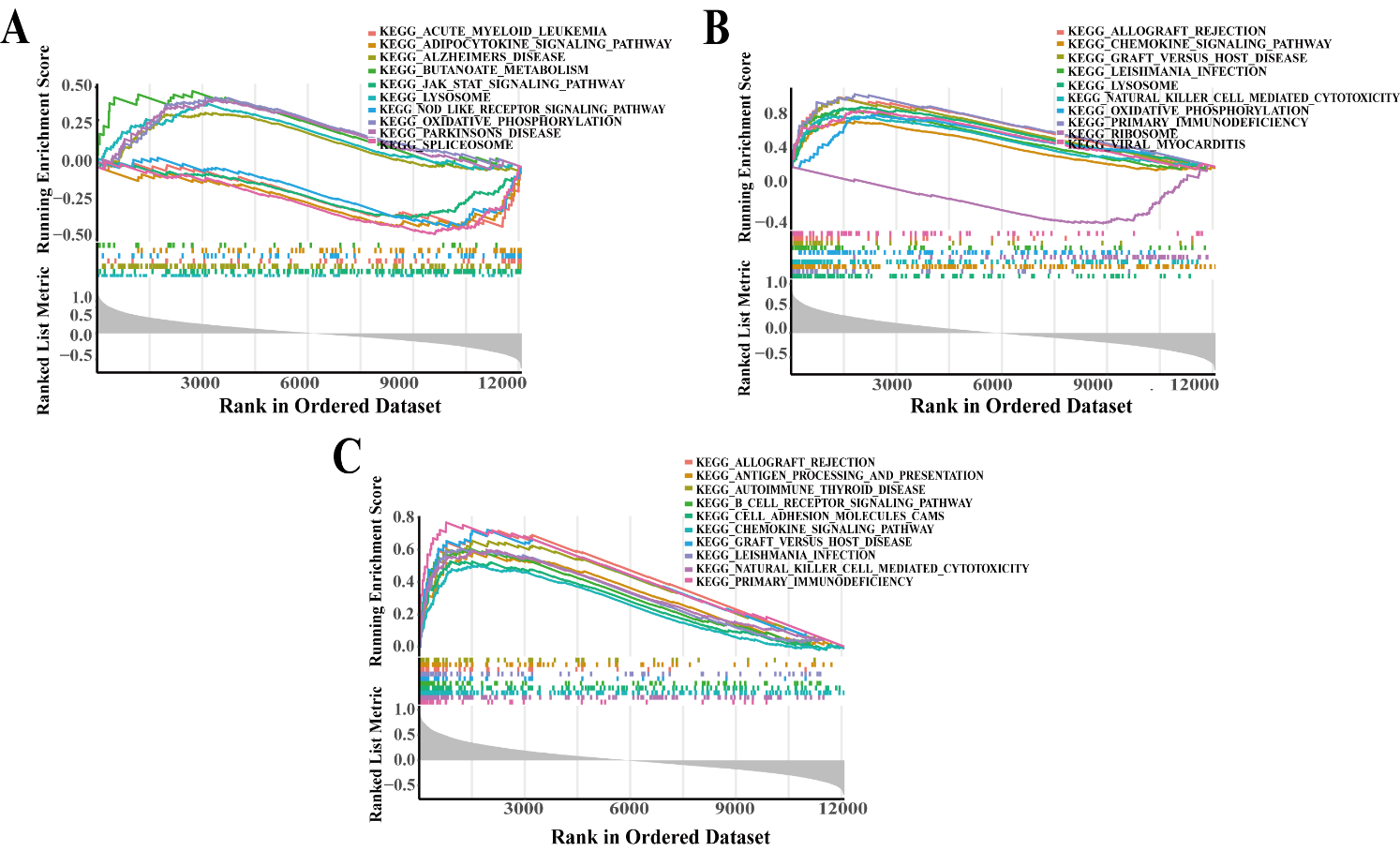


**Figure S3. Single-Gene Set Enrichment Analysis of Hub Genes.** (A-C) GSEA plot of KEGG gene sets for CX3CR1, ITGB2 and PTPRC.


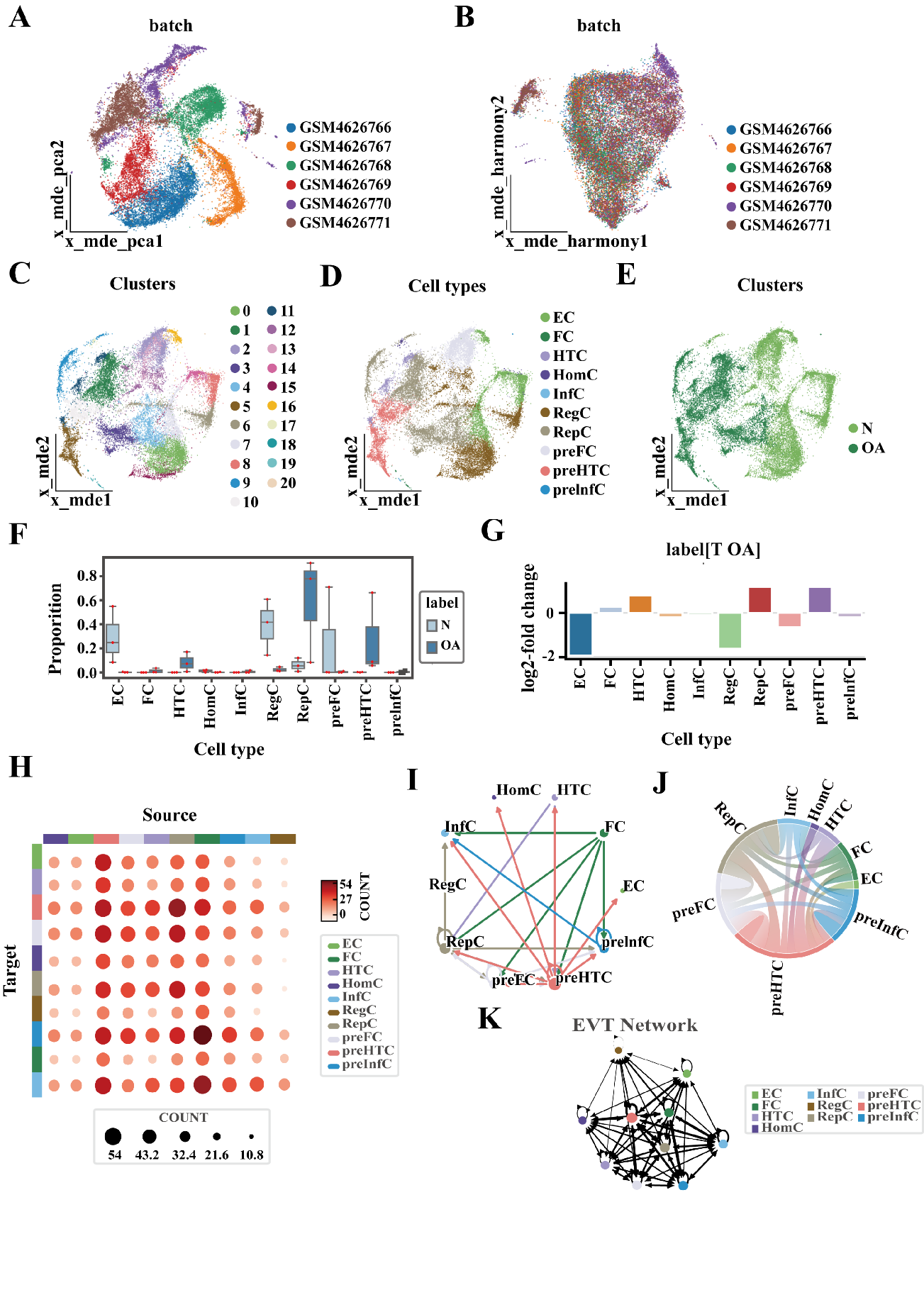


**Figure S4. Single-Cell Sequencing of Chondrocytes in Three OA Patients and Three Normal Samples.**(A) Pre-batch effect removal of six samples; (B) Post-batch effect removal of six samples; (C) Clustering of chondrocytes into 20 clusters; (D) Annotated clustering of chondrocytes; (E, F, G) UMAP distribution plots for OA and normal samples; (H) Heatmap of intercellular interactions among different subpopulations; (I, J) Chord diagrams of intercellular interactions among different subpopulations; (K) Network plot of intercellular interactions among different subpopulations.


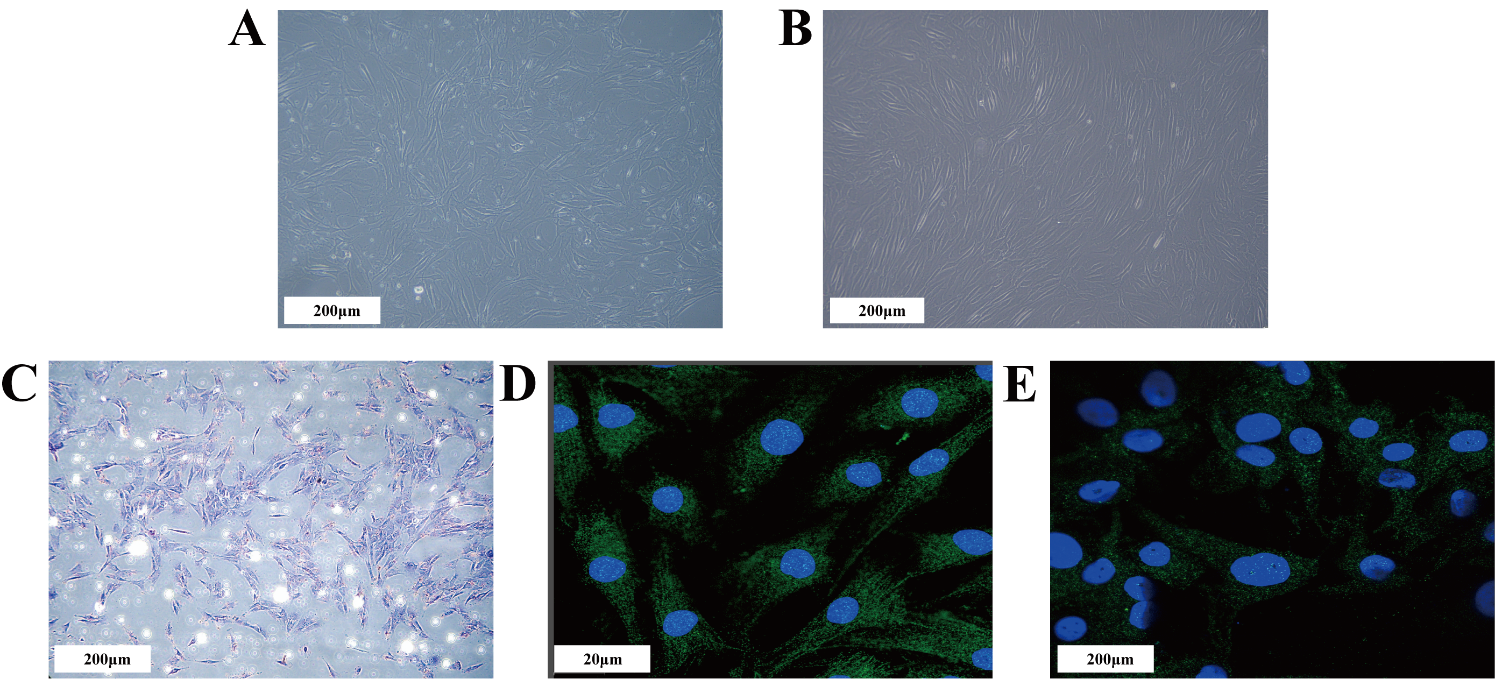


**Figure S5. Identification of chondrocytes.**

(A) Microscopic morphology of normal human chondrocytes; (B) Microscopic morphology of human OA chondrocytes; (C) Toluidine blue staining of chondrocytes; (D) Immunofluorescence staining for Collagen II in normal cells; (E) Immunofluorescence staining for Collagen II in OA cells.


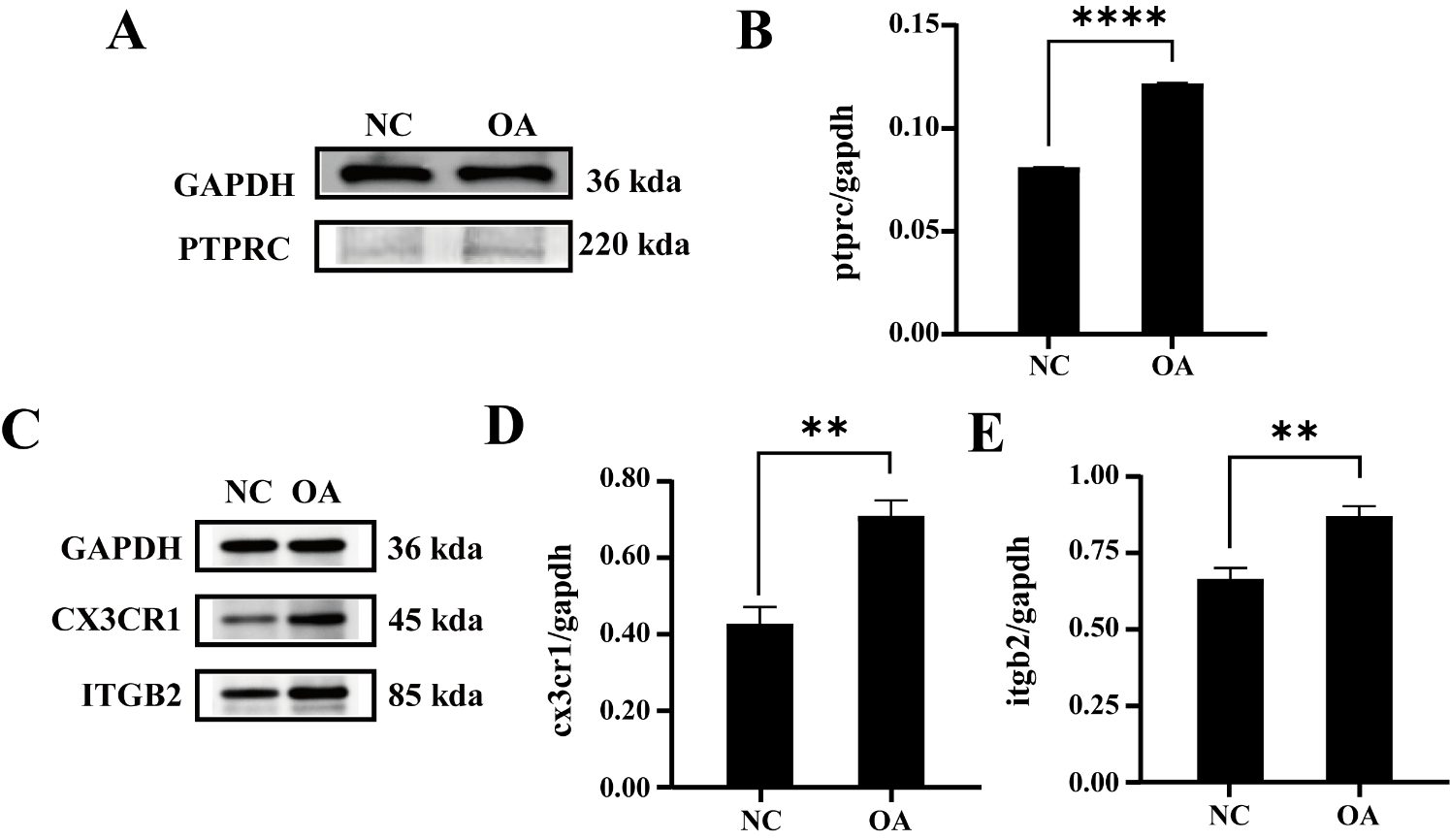


**Figure S6. Western blot validation of PTPRC, CX3CR1, and ITGB2 protein expression in OA and NC samples.**  (A) Representative WB image showing PTPRC protein levels in NC and OA cartilage samples, with GAPDH as the loading control; (B) Quantification of PTPRC protein expression normalized to GAPDH. Data are presented as mean ± SEM; (C) Representative WB images of CX3CR1 and ITGB2 protein levels in NC and OA cartilage samples, with GAPDH as the loading control; (D) Quantification of CX3CR1 protein expression normalized to GAPDH. Data are presented as mean ± SEM; (E) Quantification of ITGB2 protein expression normalized to GAPDH. Data are presented as mean ± SEM.

Note: * indicates p<0.05, ** indicates p< 0.01, **** indicates p< 0.0001, n=3.


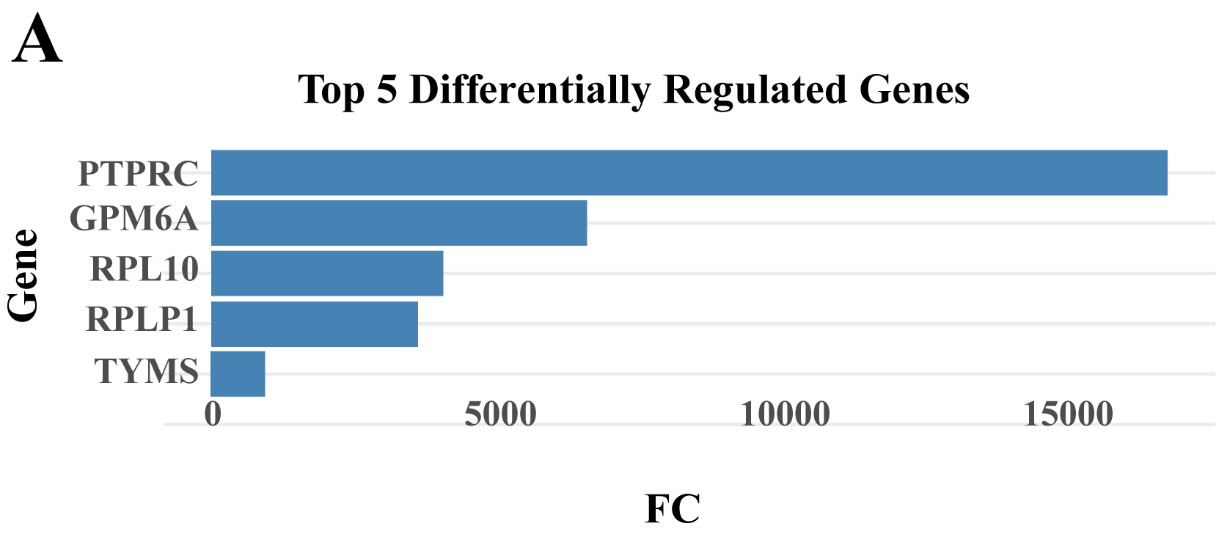


**Figure S7. Genetic disruption caused by PTPRC knockout in NK cells.**

**Table S1. Characteristics of study patients**

|  |  | Source | Patients | M/F | Age | BMI |
| --- | --- | --- | --- | --- | --- | --- |
| bioinformatics section | GSE46750 | synovial regions | Normal areas in the same OA patient | 2/10 | 50–83 |  |
|  | GSE55235 | synovial regions | Control group | 5/5 | 54.7 ± 4.0 |  |
|  |  |  | OA | 5/5 | 71.0 ± 1.4 |  |
|  | GSE169077 | synovial regions | Normal areas in the same OA patient |  |  |  |
|  | GSE98918 | meniscus | Control group | 5/7 | 49.1 ± 10 | 26.90 ± 3.91 |
|  |  |  | OA | 9/3 | 65.2± 7.94 | 36.27 ± 6.61 |
| cellular experiments |  |  | Normal areas in the same OA | 1/2 | 66±5 | 29± 3 |

|  | Age | KL grade | BMI (kg/m²) | WOMAC |
| --- | --- | --- | --- | --- |
| Female1 | 66 | 2 | 26 | 30 |
| Female2 | 71 | 4 | 29 | 49 |
| Male1 | 65 | 3 | 30 | 45 |

**Table S2. Screening of 2,022 DEGs in OA.**

**Table S3. Construction of Weighted Correlation Network for OA and Identification of 1,686 co-expressed genes**

**Table S4. Top 20 genes in PPI Network**

| Rank | Name | Score |
| --- | --- | --- |
| 1 | CD4 | 5.71669791854E11 |
| 2 | PTPRC | 5.71649316498E11 |
| 3 | CCR5 | 5.70511629602E11 |
| 4 | CCL5 | 5.60418155958E11 |
| 5 | ITGB2 | 5.56105304647E11 |
| 6 | CSF1R | 5.50368542258E11 |
| 7 | CCR2 | 5.46224587584E11 |
| 8 | SELL | 5.12785688887E11 |
| 9 | CCR1 | 5.06688155407E11 |
| 10 | CASP1 | 4.82761951664E11 |
| 11 | MMP9 | 4.79696717817E11 |
| 12 | TYROBP | 4.52732850784E11 |
| 13 | TLR8 | 3.97805761035E11 |
| 14 | CX3CR1 | 3.27055627724E11 |
| 15 | IL15 | 3.0772097496E11 |
| 16 | CYBB | 2.33184871673E11 |
| 17 | TLR1 | 2.08545581448E11 |
| 18 | CXCL12 | 1.11908032116E11 |
| 19 | VCAM1 | 1.09969289832E11 |
| 20 | CD48 | 4.5575544768E10 |

**Table S5. Mendelian randomization results for CX3CR1 and PTPRC.**

**Table S5.1** Information of identified SNPs in exposure (CX3CR1) and outcomes (OA).

|  | | | | **Exposure (CX3CR1)** | | |
| --- | --- | --- | --- | --- | --- | --- |
|  | **SNP** | **EA** | **OA** | **β** | **SE** | ***p* value** |
| 1 | rs112450242 | T | C | -1.257 | 0.2349 | 9.19E-08 |
| 2 | rs149100 | G | T | -0.121 | 0.02458 | 8.87E-07 |
| 3 | rs1801274 | G | A | -0.2388 | 0.02599 | 6.57E-20 |
| 4 | rs58215329 | T | C | 0.1903 | 0.03826 | 6.86E-07 |
| 5 | rs9877538 | A | C | -0.1766 | 0.03298 | 9.19E-08 |

| **Outcome (OA)** | | | | |
| --- | --- | --- | --- | --- |
| **Case** | **Control** | **β** | **SE** | ***p* value** |
| 149337 | 262844 | -0.0454085 | 0.0159624 | 0.00444519 |
| 149337 | 262844 | -0.00343773 | 0.00652911 | 0.598525 |
| 149337 | 262844 | -0.00496932 | 0.00615603 | 0.419535 |
| 149337 | 262844 | 0.0251416 | 0.0120417 | 0.0368078 |
| 149337 | 262844 | 0.00758706 | 0.0080484 | 0.345845 |

**Table S5.2** Information of identified SNPs in exposure (PTPRC) and outcomes (OA).

|  |  | | | **Exposure (PTPRC)** | | |
| --- | --- | --- | --- | --- | --- | --- |
|  | **SNP** | **EA** | **OA** | **β** | **SE** | ***p* value** |
| 1 | rs11141907 | G | A | -0.5593 | 0.1229 | 5.55E-06 |
| 2 | rs113853911 | C | G | -0.2456 | 0.05234 | 2.81E-06 |
| 3 | rs117509040 | C | G | 1.413 | 0.3163 | 8.17E-06 |
| 4 | rs12131677 | A | G | 0.124 | 0.02762 | 7.33E-06 |
| 5 | rs12364299 | T | A | -0.2989 | 0.05053 | 3.68E-09 |
| 6 | rs142767469 | A | T | -0.7064 | 0.1376 | 3.01E-07 |
| 7 | rs182879147 | A | T | -1.115 | 0.229 | 1.18E-06 |
| 8 | rs2511241 | T | C | 0.3515 | 0.07011 | 5.62E-07 |
| 9 | rs267708 | G | A | 0.1269 | 0.02804 | 6.28E-06 |
| 10 | rs34858919 | A | C | -0.1393 | 0.02873 | 1.31E-06 |
| 11 | rs58552272 | C | A | -0.2054 | 0.04543 | 6.39E-06 |
| 12 | rs6884558 | G | A | 0.12 | 0.02659 | 6.56E-06 |
| 13 | rs7005676 | T | C | 0.1801 | 0.03743 | 1.56E-06 |
| 14 | rs7323586 | C | G | -0.1432 | 0.03051 | 2.82E-06 |
| 15 | rs74798265 | G | C | 0.5665 | 0.1191 | 2.07E-06 |
| 16 | rs77687616 | C | T | -0.7414 | 0.1375 | 7.55E-08 |
| 17 | rs79265501 | T | C | -0.2792 | 0.05782 | 1.44E-06 |
| 18 | rs867396 | C | G | 0.1216 | 0.02687 | 6.31E-06 |

| **Outcome (OA)** | | | | |
| --- | --- | --- | --- | --- |
| **Case** | **Control** | **β** | **SE** | ***p* value** |
| 149337 | 262844 | -0.00980513 | 0.0207318 | 0.636247 |
| 149337 | 262844 | 0.00831738 | 0.0243205 | 0.732359 |
| 149337 | 262844 | 0.0197768 | 0.0233198 | 0.3964 |
| 149337 | 262844 | -0.00553071 | 0.00829534 | 0.504948 |
| 149337 | 262844 | -0.0270184 | 0.0210207 | 0.198679 |
| 149337 | 262844 | 0.00971427 | 0.0133353 | 0.46633 |
| 149337 | 262844 | -0.0241274 | 0.01538 | 0.116706 |
| 149337 | 262844 | 0.0120094 | 0.0115822 | 0.299789 |
| 149337 | 262844 | 0.0017666 | 0.00702367 | 0.801412 |
| 149337 | 262844 | -0.00209936 | 0.00699001 | 0.76392 |
| 149337 | 262844 | 0.00292494 | 0.0129175 | 0.820865 |
| 149337 | 262844 | 0.00459225 | 0.00618527 | 0.457815 |
| 149337 | 262844 | 0.00936626 | 0.0125399 | 0.455115 |
| 149337 | 262844 | -0.00769376 | 0.00644554 | 0.232612 |
| 149337 | 262844 | 0.0401036 | 0.0178477 | 0.0246411 |
| 149337 | 262844 | -0.0116039 | 0.0399213 | 0.771303 |
| 149337 | 262844 | 0.0087949 | 0.0308154 | 0.775333 |
| 149337 | 262844 | -0.00925123 | 0.00634906 | 0.145089 |

**Table S5.3** the results of Mendelian randomization analysis.

| **Exposure** | **Outcome** | **MR method** | **OR** | **95%CI** | **P value** |
| --- | --- | --- | --- | --- | --- |
| CX3CR1 | OA | MR Egger | 1.039 | 1.000 to 1.079 | 0.144 |
| CX3CR1 | OA | Weighted median | 1.034 | 1.005 to 1.062 | 0.018 |
| CX3CR1 | OA | Inverse variance weighted | 1.032 | 1.007 to 1.058 | 0.012 |
| CX3CR1 | OA | Simple mode | 1.029 | 0.987 to 1.072 | 0.244 |
| CX3CR1 | OA | Weighted mode | 1.036 | 1.009 to 1.062 | 0.053 |
| PTPRC | OA | MR Egger | 1.015 | 0.994 to 1.036 | 0.164 |
| PTPRC | OA | Weighted median | 1.017 | 0.994 to 1.040 | 0.131 |
| PTPRC | OA | Inverse variance weighted | 1.018 | 1.002 to 1.033 | 0.019 |
| PTPRC | OA | Simple mode | 1.020 | 0.984 to 1.057 | 0.284 |
| PTPRC | OA | Weighted mode | 1.017 | 0.996 to 1.039 | 0.124 |

The causal effect between CX3CR1, PTPRC and OA was evaluated

**Table S5.4** the results of MR-Egger's intercept analysis.

| **Exposure** | **Outcome** | **Egger_intercept** | **SE** | ***p* value** |
| --- | --- | --- | --- | --- |
| CX3CR1 | OA | -0.003250513 | 0.006621489 | 0.657161389 |
| PTPRC | OA | 0.0011868707 | 0.003590290 | 0.745535676 |

**Table S5.5** the results of heterogeneity analysis.

| **Exposure** | **Outcome** | **Method** | **Q** | **Q_df** | **Q_*p* val** |
| --- | --- | --- | --- | --- | --- |
| CX3CR1 | OA | MR Egger | 5.0962415 | 3 | 0.164883999 |
| CX3CR1 | OA | Inverse variance weighted | 5.5056167 | 4 | 0.239236208 |
| PTPRC | OA | MR Egger | 9.6030627 | 15 | 0.843938071 |
| PTPRC | OA | Inverse variance weighted | 9.7123445 | 16 | 0.881212365 |
